# Modification‐Response Integrated Modules Driven Cyclization‐Dependent Prodrug Self‐Assembly for Reshaping Pro‐Apoptotic Tumor Redox Homeostasis

**DOI:** 10.1002/EXP.20240448

**Published:** 2026-03-10

**Authors:** Yixin Sun, Shiyi Zuo, Wenfeng Zang, Lingxiao Li, Xianbao Shi, Mingzhong Li, Zhonggui He, Bingjun Sun, Jin Sun

**Affiliations:** ^1^ Department of Pharmaceutics Wuya College of Innovation Shenyang Pharmaceutical University Shenyang China; ^2^ Department of Pharmacy The First Affiliated Hospital of Jinzhou Medical University Jinzhou China; ^3^ Leicester School of Pharmacy De Montfort University Leicester UK; ^4^ Joint International Research Laboratory of Intelligent Drug Delivery Systems Ministry of Education，Shenyang Pharmaceutical University Shenyang China

**Keywords:** cancer therapy, cyclic chalcogenides, modification‐response integrated modules, prodrug nanoassemblies, tumor redox homeostasis

## Abstract

Affected by the complexity and heterogeneity of the tumor redox microenvironment, chemotherapy often fails to achieve satisfactory clinical outcomes. Modular design of prodrug nanoassemblies presents tremendous potential in upgrading the therapeutic index of chemotherapeutic agents. Given the biochemical vulnerability of tumor redox homeostasis, we fabricated five‐membered cyclic chalcogenide‐linked paclitaxel prodrug nanoassemblies, which realized tumor site‐specific activation and reshaped the pro‐apoptotic tumor redox homeostasis. Cyclic diselenide and cyclic disulfide bonds integrated the modification modules and response modules to minimize the utilization of non‐pharmacodynamic moieties and increase druggability. Importantly, the modification‐response integrated modules could simultaneously block the glutathione‐glutathione peroxidase (GSH/GPx) antioxidant system and amplify reactive oxygen species (ROS) generation. The reshaping of the tumor redox homeostasis cascade triggered the loss of mitochondrial membrane potential and apoptosis of tumor cells, which synergistically potentiated the antitumor effects of paclitaxel. Such an intelligent prodrug nanoplatform brought new perspectives for constructing advanced antitumor nanomedicines with significant clinical research value.

## Graphical abstract

1. The concept of modification‐response integrated modules was proposed for the first time.

2. Simple, efficient, and feasible drug delivery platforms were developed for systematic regulation of tumor redox microenvironment and reshaping of pro‐apoptotic tumor redox homeostasis.

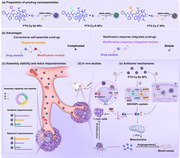

## 1 Introduction

The complexity of the tumor microenvironment remains a challenge for chemotherapy [1, 2]. The construction of intelligent drug delivery systems (DDS s) is one of the mainstream strategies to overcome this drawback [3, 4, 5, 6, 7]. Oncogenic factors, such as the activation of oncogenes and alterations in mitochondrial activity, promote the generation of ROS within cancer cells [8]. To compensate for the excessive accumulation of ROS, cancer cells utilize the antioxidant system to elevate the level of glutathione (GSH) to maintain redox homeostasis. Consequently, cancer cells display higher ROS and GSH levels than normal cells. Due to the remarkable discrepancy in redox levels from tumor cells to normal cells, redox‐responsive DDSs have attracted extensive attention [9, 10, 11, 12]. However, most redox‐responsive DDSs can only respond but fail to interfere with tumor redox homeostasis, restricting the effectiveness of chemotherapy.

Intracellular redox homeostasis, a dynamic balance of ROS production and scavenging, is the cornerstone for maintaining cellular physiological steady state [13, 14]. The biochemical vulnerability of high‐level redox homeostasis in tumor cells has emerged as a hotspot for effective tumor therapy [15]. When tumor cells are exposed to harmful stimuli (e.g., chemotherapy or radiotherapy), the excessive production of ROS exceeds the scavenging capacity of the antioxidant system, thereby tilting intracellular redox homeostasis toward oxidative stress and inducing tumor cell death [16, 17, 18]. However, the glutathione‐glutathione peroxidase (GSH/GPx) antioxidant system features a high expression in tumor cells to resist oxidative stress. Glutathione (GSH), glutathione reductase (GR), and glutathione peroxidase (GPx) are the three crucial components of the GSH/GPx system. GSH, an important small molecule non‐protein thiol, plays a critical role in scavenging free radicals. GR catalyzes the reduction of oxidized GSH (GSSG) to GSH and regulates the production of GSH [19]. GPx resists oxidative injury by catalyzing the decomposition of hydrogen peroxide (H_2_O_2_, a species of ROS). Therefore, therapeutic strategies that simultaneously block the GSH/GPx system and promote ROS generation can synergistically disrupt tumor redox homeostasis and exacerbate tumor cell apoptosis.

Prodrug nanoassemblies, which are obtained by self‐assembly of prodrugs, combine the merits of prodrug strategies and nanomedicines. We are committed to the study of redox‐responsive prodrug nanoassemblies [20, 21, 22, 23]. Briefly, the prodrugs are typically formed by drug module, response module, and modification module. The response modules govern the activation of prodrugs, which is essential for potency and safety. In addition, the inherent space structure and intermolecular forces of response modules also affect the assembly behavior of prodrugs [24, 25, 26, 27]. The modification modules facilitate the self‐assembly of the prodrugs. However, there are two bottlenecks in the modular design: i) the commonly used modification modules are non‐pharmacodynamic moieties that may pose safety risks, decreasing the druggability of prodrug nanoassemblies; ii) the ternary system constructed by the drug module, response module, and modification module increases the complexity of the structure‐activity relationship.

**SCHEME 1:**
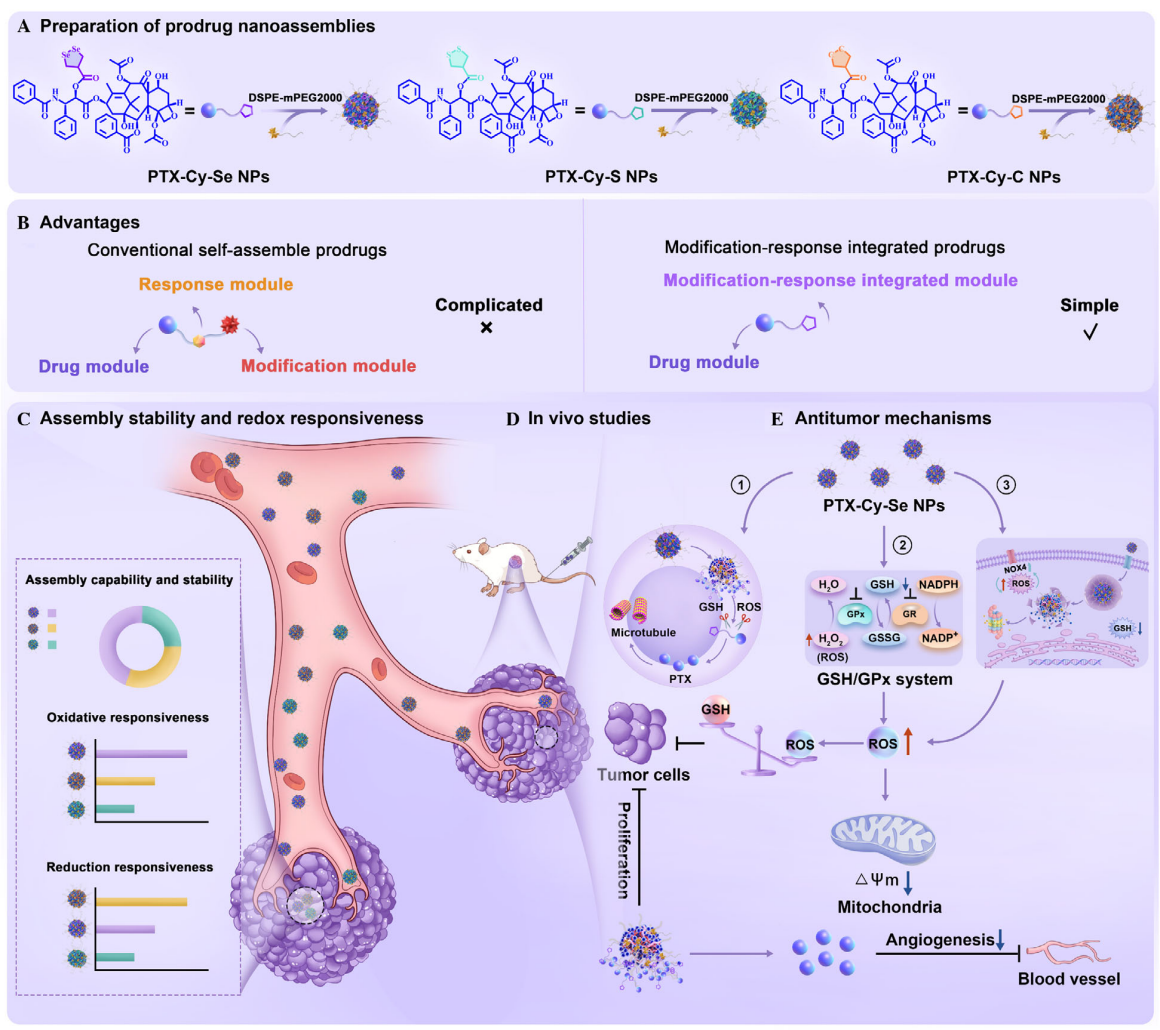
Schematic illustration. Modification‐response integrated modules drove the stable assembly of prodrug nanoassemblies and reshaped the pro‐apoptotic tumor redox homeostasis.

Herein, to address the above issues, five‐membered cyclic modules were developed, which integrated the response module and the modification module into one system. The prodrug nanoassemblies were constructed by linking 1,2‐diselenolane‐4‐carboxylic acid (Cy‐Se), 1,2‐dithiolane‐4‐carboxylic acid (Cy‐S), or cyclopentanecarboxylic acid (Cy‐C) to paclitaxel (PTX). Compared to conventional self‐assembly prodrugs, the integration of the response module and modification module in a five‐membered ring significantly reduced the complexity of the prodrugs, which minimized the use of non‐pharmacological moieties from the source of structure design. Interestingly, the cyclic diselenide module not only ensured the stable assembly of the prodrug but also realized the responsive modulation of tumor redox homeostasis. In particular, the prodrug with a cyclic diselenide module possessed the best assembly stability due to the strong chalcogen bonding (intermolecular force) and the suitable bond angle (steric hindrance). More importantly, the prodrug nanoassemblies systematically modulated the tumor redox homeostasis and induced apoptosis in multiple pathways, including preventing microtubule protein depolymerization, blocking the GSH/GPx system, inducing ROS overproduction, and causing loss of mitochondrial membrane potential (Scheme 1).

## 2 Results and Discussion

### 2.1 Fabrication and Characterization of Prodrug Nanoassemblies

Three unreported prodrugs were synthesized as illustrated in Supplementary Figure S1. First, the 1,2‐diselenolane‐4‐carboxylic acid intermediate was synthesized. Then, PTX was conjugated with 1,2‐diselenolane‐4‐carboxylic acid, 1,2‐dithiolane‐4‐carboxylic acid, or cyclopentanecarboxylic acid by esterification reaction to synthesize PTX‐Cy‐Se, PTX‐Cy‐S, or PTX‐Cy‐C. The successful synthesis of three prodrugs was verified using high‐resolution mass spectrometry (HRMS), ^1^H nuclear magnetic resonance spectroscopy (^1^H NMR), and high performance liquid chromatography ((HPLC), Supplementary Figures S2–S4).

**FIGURE 1:**
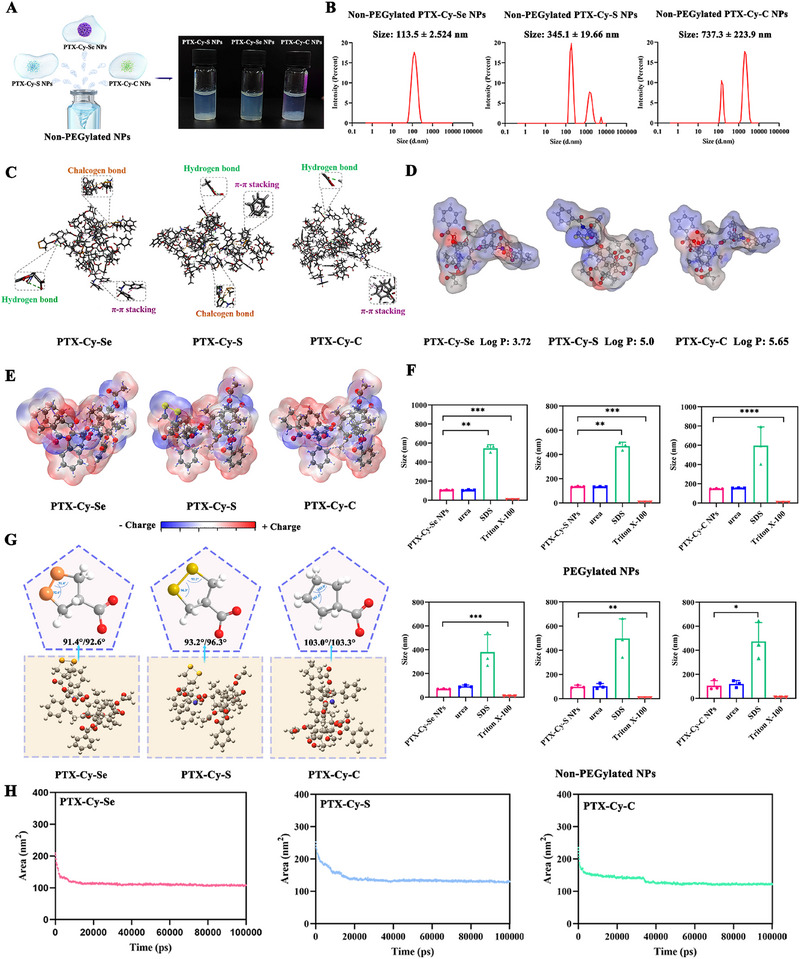
Exploration of the assembly mechanism. (A) The appearance of non‐PEGylated PTX‐Cy‐Se NPs, PTX‐Cy‐S NPs, and PTX‐Cy‐C NPs (0.6 mg mL^−1^). (B) Particle size of non‐PEGylated PTX‐Cy‐Se NPs, PTX‐Cy‐S NPs, and PTX‐Cy‐C NPs (0.6 mg mL^−1^),. (C) Intermolecular forces of prodrugs.  (D) The oil‐water partition coefficient of PTX‐Cy‐Se, PTX‐Cy‐S, and PTX‐Cy‐C,. (E) Electrostatic potential map of PTX‐Cy‐Se, PTX‐Cy‐S, and PTX‐Cy‐C. (F) The particle size changes of PEGylated NPs and non‐PEGylated NPs after treatment with 0.4 M of urea, SDS, and Triton X‐100. (G) Bond angles of ‐C‐Se‐Se‐C, ‐C‐S‐S‐C, and ‐C‐C‐C‐C in prodrugs, and (H) SASA of three prodrugs for molecular simulations at 0–100,000 ps.

Next, a one‐step nanoprecipitation method was used to prepare nanoassemblies. Three prodrugs self‐assembled into non‐PEGylated nanoparticles in deionized water (Supplementary Figure S5, Supplementary Table S1, 0.2 mg mL^−1^). As the prodrug concentration increased, precipitation and enlarged particle size were observed for PTX‐Cy‐S NPs and PTX‐Cy‐C NPs (Figures 1A, B, 0.6 mg mL^−1^). Interestingly, no significant precipitation and particle size variation occurred in PTX‐Cy‐Se NPs, suggesting that the five‐membered cyclic diselenide bond was more conducive to stable self‐assembly of the prodrugs. To explore this reason, molecular dynamics simulations and force disruption experiments were carried out. The presence of intermolecular π‐π stacking, hydrophobic forces, hydrogen bonding, and chalcogen bonding (ChB) contributed to prodrug assembly (Figures 1C, D). ChB was the interaction between the positively polarized chalcogen atom and the Lewis base, leading to the appearance of σ‐hole (a region of positive electrostatic potential) [28]. Notably, ChB was observed in the intermolecular forces of PTX‐Cy‐Se and PTX‐Cy‐S. Compared to S, the σ‐hole of Se was more positive, and Se was more polarizable, which offered the possibility to generate a stronger ChB interaction than that of S [29]. Furthermore, the charge distribution of PTX‐Cy‐Se and PTX‐Cy‐S was more uniform and balanced (Figure 1E). Then, we conducted proof‐of‐concept experiments to demonstrate that hydrophobic forces (disrupted by Triton X‐100 and SDS) and hydrogen bonding (disrupted by urea) drove the self‐assembly of the prodrugs (Figure 1F).

Notably, the balance between intermolecular forces and steric hindrances was essential to the aggregation or dispersion of nanoassemblies [30]. Excessive intermolecular forces induced the aggregation of nanoassemblies and poor colloidal stability. Therefore, the suitable steric hindrance was also an important guarantee for the stability of nanoassemblies. It was documented that the introduction of sulfur‐containing bond angles approaching 90° could modulate the steric hindrances and increase the structural flexibility of prodrugs, which promoted the self‐assembly of prodrugs [31]. After geometry optimization, the bond angles of ‐C‐Se‐Se‐C (91.4°/92.6°), ‐C‐S‐S‐C (93.2°/96.3°), and ‐C‐C‐C‐C (103.0°/103.3°) in the five‐membered ring of the prodrugs were calculated (Figure 1G) [32, 33]. Importantly, the bond angle of ‐C‐Se‐Se‐C (91.4°/92.6°) was closest to 90°, which was more favorable for increasing the structural flexibility of the prodrugs to form more stable nanoassemblies. In addition, the solvent‐accessible surface area (SASA) of PTX‐Cy‐Se (100,000 ps = 107.77 nm^2^) was smaller than that of PTX‐Cy‐S (100,000 ps = 129.68 nm^2^) and PTX‐Cy‐C (100,000 ps = 121.51 nm^2^), suggesting that PTX‐Cy‐Se might form more compact nanostructures (Figure 1H). This was consistent with the room temperature stability of the non‐PEGylated PTX‐Cy‐Se NPs, PTX‐Cy‐S NPs, and PTX‐Cy‐C NPs (Supplementary Figure S6).

**FIGURE 2:**
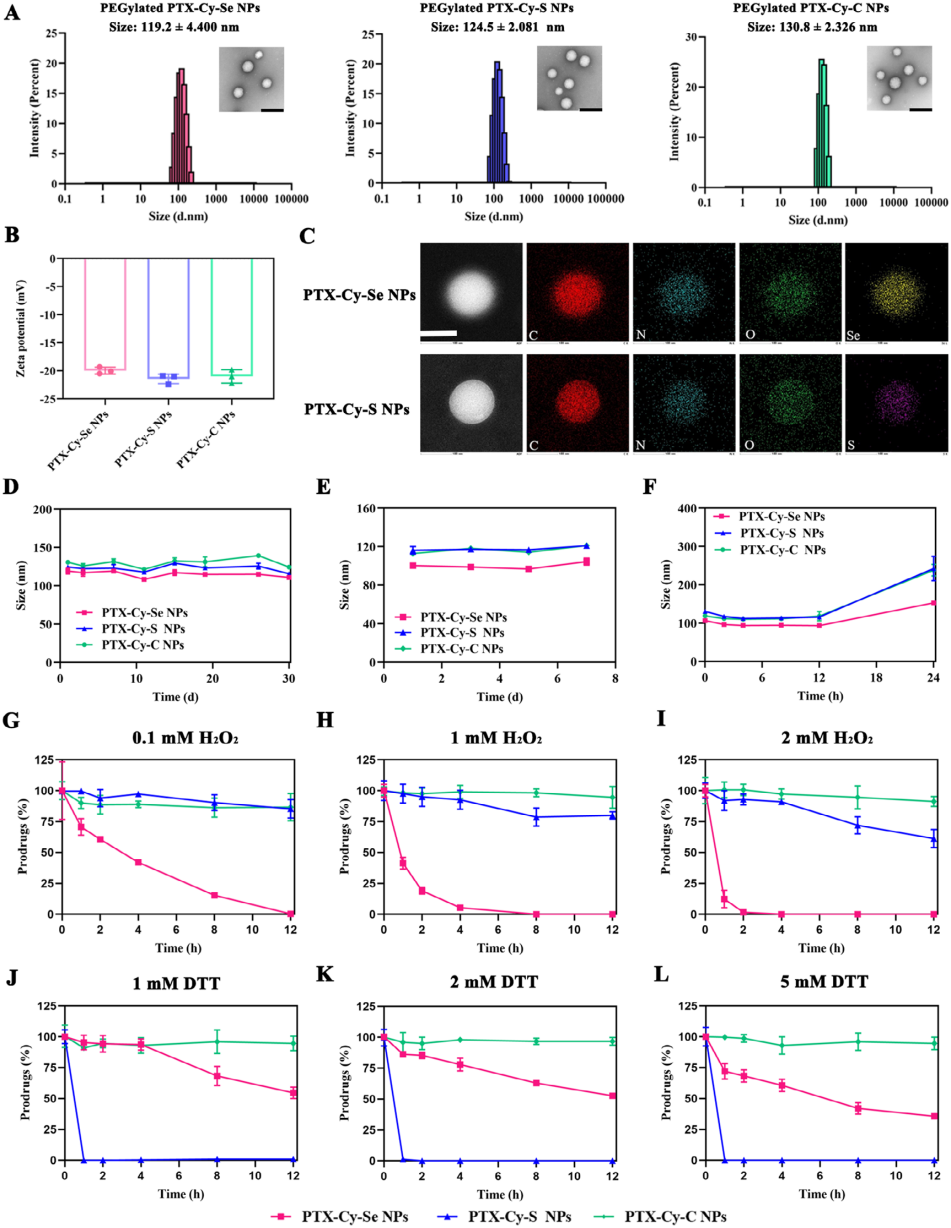
Characterization and redox responsiveness of prodrug nanoassemblies. (A) Particle size distribution, and TEM images (scale bars: 200 nm), (B) Zeta potential, and (C) element mapping (scale bar: 100 nm) of prodrug nanoassemblies (1 mg mL^−1^). Long‐term storage stability of PTX‐Cy‐Se NPs, PTX‐Cy‐S NPs, and PTX‐Cy‐C NPs at (D) 4°C for 30 days and (E) room temperature for 7 days. (F) Colloidal stability of prodrug nanoassemblies in PBS containing 10% fetal bovine serum. Activation profiles of prodrug nanoassemblies in release media containing (G) 0.1 mM H_2_O_2_ (*n* = 3), (H) 1 mM H_2_O_2_ (*n* = 3), (I) 2 mM H_2_O_2_ (*n* = 3), (J) 1 mM DTT (*n* = 3), (K) 2 mM DTT (*n* = 3), and (L) 5 mM DTT (*n* = 3).

Next, DSPE‐mPEG_2000_ (20%, w/w) was utilized to increase the colloidal stability and decrease protein adsorption. For PEGylated NPs, three prodrug nanoassemblies displayed spherical morphology, featuring particle sizes around 120 nm and surface zeta potential of about ‐20 mV (Figures 2A, B and Supplementary Table S2). Uniform distribution of different elements in the prodrug nanoassemblies was observed in the elemental mapping images, especially S and Se elements (Figure 2C). Dual role of prodrugs as nanocarriers and payloads resulted in ultra‐high drug‐loading of the prodrug nanoassemblies (63.18% for PTX‐Cy‐Se, 69.33% for PTX‐Cy‐S and 71.95% for PTX‐Cy‐C). Moreover, the prodrug nanoassemblies exhibited satisfactory long‐term storage stability (Figures 2D, E). As shown in Figure 2F, PTX‐Cy‐Se NPs had better colloidal stability than PTX‐Cy‐S NPs and PTX‐Cy‐C NPs due to the strong intermolecular forces and the optimal selenium‐containing bond angles.

### 2.2 Activation Mechanism of Redox‐Responsive Prodrug Nanoassemblies

**FIGURE 3:**
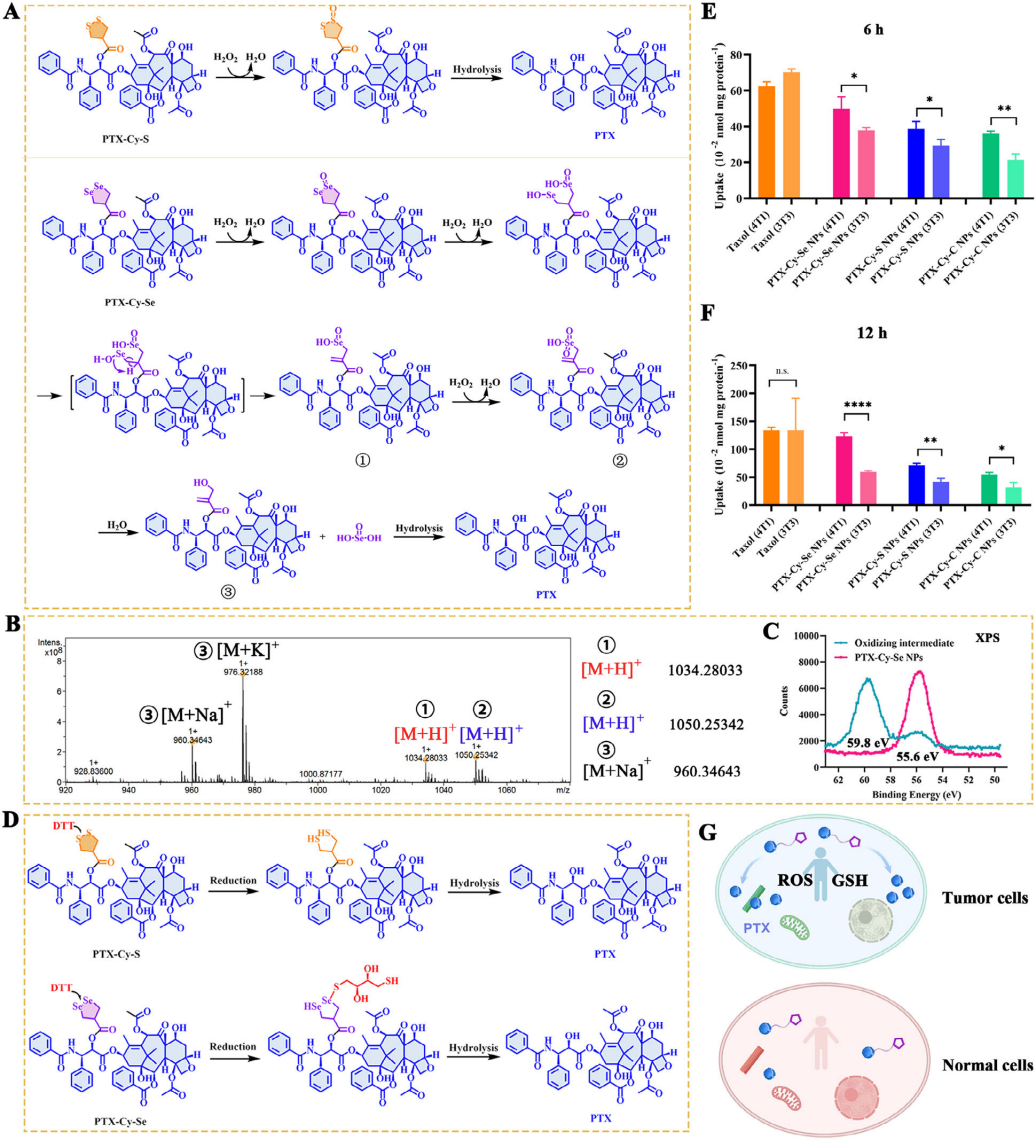
Redox activation mechanisms and cellular activation of prodrug nanoassemblies. (A) The oxidative activation mechanism of PTX‐Cy‐S NPs and PTX‐Cy‐Se NPs. (B–C) Mass spectrum and XPS analysis of the oxidation intermediate of PTX‐Cy‐Se NPs under 1 mM H_2_O_2_. (D) Mechanism of reductive activation of PTX‐Cy‐S NPs and PTX‐Cy‐Se NPs. The released PTX from PTX‐Cy‐Se NPs, PTX‐Cy‐S NPs, and PTX‐Cy‐C NPs (10 µM) after (E) 6 h and (F) 12 h of cellular uptake (*n* = 3). (G) Schematic diagram of tumor‐selective drug release of prodrug nanoassemblies.

As far as we know, the difference between the redox responsiveness of the cyclic diselenide bond and the cyclic disulfide bond was unclear. Here, we explored the in vitro activation behavior of PTX‐Cy‐Se NPs, PTX‐Cy‐S NPs, and PTX‐Cy‐C NPs and the intracellular release of PTX. Under oxidizing conditions, the oxidative responsiveness was PTX‐Cy‐Se NPs > PTX‐Cy‐S NPs > PTX‐Cy‐C NPs (Figures 2G–I). The higher the concentration of hydrogen peroxide (H_2_O_2_), the faster the degradation of the prodrug nanoassemblies. The oxidative activation mechanism was investigated in detail. The cyclic disulfide bond was attacked by H_2_O_2_ to generate a monoxide, which enhanced the hydrophilicity and accelerated the hydrolysis of the adjacent ester bond (Figure 3A and Supplementary Figure S7). Compared with the cyclic disulfide bond, the cyclic diselenide bond was more easily oxidized due to its larger atomic radius and lower bond energy (Figure 3A). Interestingly, the ‐1 valent diselenide bond was first oxidized to monoxide, which could be further oxidized to selenenic acid and seleninic acid. The selenenic acid underwent the intramolecular selenoxide elimination reaction to generate the acrylate‐like structure. Meanwhile, seleninic acid was further oxidized to selenonic acid, which suffered from the hydrolysis reaction to produce the final intermediate. The oxidation intermediates were corroborated by HRMS and ^1^H‐NMR (Figure 3B and Supplementary Figure S8). After the oxidation of PTX‐Cy‐Se NPs, a peak of 59.8 eV appeared in X‐ray photoelectron spectroscopy (XPS) analysis (Figure 3C), which could be classified as +4 valence selenium [34]. The XPS spectrum further supported the oxidation process of the five‐membered cyclic diselenide bond.

Under reducing conditions, the reducing responsiveness of prodrug nanoassemblies was PTX‐Cy‐S NPs > PTX‐Cy‐Se NPs > PTX‐Cy‐C NPs (Figures 2J–L). From the perspective of the reducing mechanism, dithiothreitol (DTT) was able to break the disulfide bond of PTX‐Cy‐S NPs to generate free thiols, thus enhancing the hydrophilicity of the intermediate and promoting the hydrolysis of the adjacent ester bond (Figure 3D and Supplementary Figure S9). Compared to the disulfide bond, the diselenide bond was more likely to exchange with the thiol of DTT to form Se‐S bonds, which only exposed one hydrophilic selenol in the intermediate (Figure 3D and Supplementary Figure S10). Therefore, the reduction responsiveness of PTX‐Cy‐Se NPs was weaker than that of PTX‐Cy‐S NPs. PTX‐Cy‐C NPs showed the lowest activation efficiency due to the absence of redox‐sensitive moieties. To investigate the selective activation of PTX‐Cy‐Se NPs, PTX‐Cy‐S NPs, and PTX‐Cy‐C NPs in tumor cells, HPLC was used to detect the released PTX in tumor cells (4T1 cells and A549 cells) and 3T3 normal cells. The quantitative results of PTX in cells were Taxol > PTX‐Cy‐Se NPs > PTX‐Cy‐S NPs > PTX‐Cy‐C NPs (Figures 3E–G, Supplementary Figure S11). For Taxol, there was no difference between tumor cells and 3T3 normal cells. However, the PTX release from the PTX‐Cy‐Se NPs, PTX‐Cy‐S NPs and PTX‐Cy‐C NPs was lower in 3T3 cells than in tumor cells, which guaranteed the potency and safety of the chemotherapy.

### 2.3 Cytotoxicity

**FIGURE 4:**
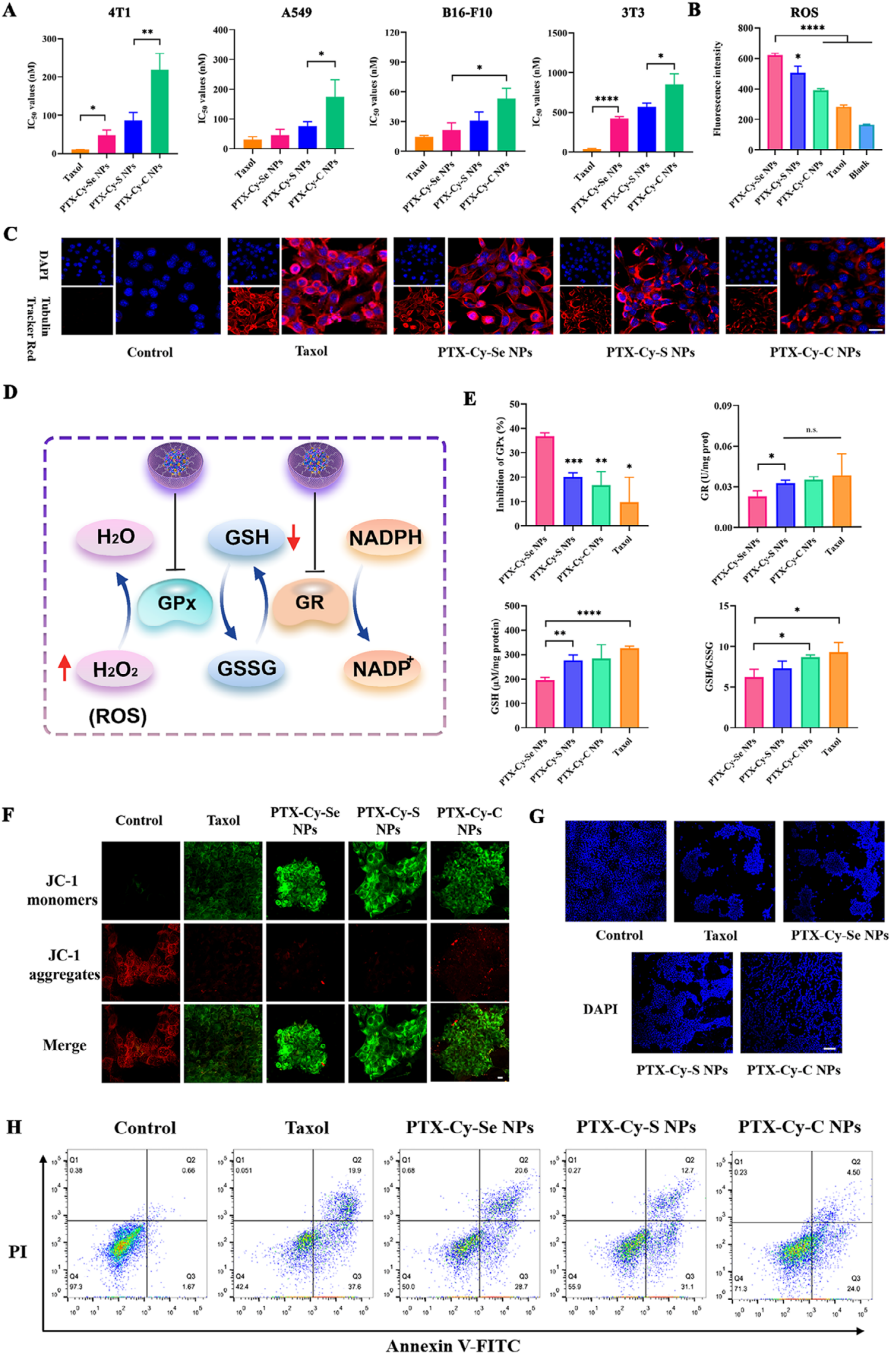
Cytotoxicity and multiple pathway‐mediated apoptosis. (A) IC_50_ values of Taxol, PTX‐Cy‐Se NPs, PTX‐Cy‐S NPs, and PTX‐Cy‐C NPs (*n* = 3). (B) Intracellular ROS generation after treatment with Taxol and prodrug nanoassemblies (*n* = 3). Effects of Taxol and prodrug nanoassemblies (100 nM, PTX equivalent) on (C) microtubulin (scale bar: 20 µm). (D–E) GR and GPx activities and GSH levels (*n* = 3), (F) mitochondrial membrane potential (scale bar: 10 µm), and (G–H) apoptosis in 4T1 cells (scale bar: 100 µm).

The in vitro antitumor activity and safety of PTX‐Cy‐Se NPs, PTX‐Cy‐S NPs and PTX‐Cy‐C NPs were evaluated by detecting the cytotoxicity against tumor cells (4T1, B16‐F10 and A549) and normal cells (3T3). According to Figure 4A and Supplementary Table S3, the half‐maximum inhibitory concentration (IC_50_) of PTX‐Cy‐Se NPs, PTX‐Cy‐S NPs and PTX‐Cy‐C NPs was lower than that of Taxol. For the prodrug nanoassemblies, the order of potency against tumor cells was PTX‐Cy‐Se NPs > PTX‐Cy‐S NPs > PTX‐Cy‐C NPs, which was in line with the PTX release in cellular uptake (Figures 3E, F). Compared with Taxol, more normal cells survived after treatment with prodrug nanoassemblies, suggesting the higher tumor‐selective cytotoxicity and superior safety of prodrug nanoassemblies (Supplementary Table S4).

### 2.4 Multiple Pathway‐Mediated Apoptosis

The antitumor activity of PTX‐Cy‐Se NPs, PTX‐Cy‐S NPs, and PTX‐Cy‐C NPs was preliminarily evaluated by cytotoxicity. Subsequently, we tried to elucidate the antitumor mechanism of PTX‐Cy‐Se NPs, PTX‐Cy‐S NPs, and PTX‐Cy‐C NPs. Firstly, it was well known that PTX killed tumor cells by inhibiting microtubule protein depolymerization. As shown in Figure 4C, 4T1 cells showed different degrees of inhibition of microtubule protein depolymerization after treatment with Taxol and prodrug nanoassemblies. Compared with PTX‐Cy‐S NPs and PTX‐Cy‐C NPs, PTX‐Cy‐Se NPs had stronger red fluorescence intensity due to more efficient intracellular PTX release, which effectively inhibited the growth of tumor cells.

Secondly, the redox homeostasis of tumor cells was more fragile compared to that of normal cells. As the prodrug nanoassemblies exhibited dual redox sensitivity and the discovery that diselenide bonds could induce ROS generation, the effect of prodrug nanoassemblies on tumor redox homeostasis was investigated. Compared with PTX‐Cy‐S NPs and PTX‐Cy‐C NPs, PTX‐Cy‐Se NPs increased the ROS levels in 4T1 cells (Figure 4B). Moreover, the antioxidant system scavenged intracellular ROS to resist the disruption of redox homeostasis. Therefore, inhibition of the antioxidant system might promote apoptosis of tumor cells. As presented in Figures 4D, E, PTX‐Cy‐Se NPs significantly inhibited the activities of GPx and GR. Inhibition of GPx reduced the decomposition of hydrogen peroxide, which further elevated the intracellular ROS level. Inhibition of GR downregulated the conversion of GSSG to GSH, which reduced the intracellular GSH level. Ultimately, the redox balance within the tumor cells was tilted toward oxidative stress.

Finally, the elevation of ROS could destroy the mitochondrial membrane and alter the membrane permeability. Then, the ion concentration difference between the inside and outside of the membrane was reduced by free diffusion, which led to the loss of membrane potential. Compared to the control, the dramatically intensified green fluorescence and reduced red fluorescence of prodrug nanoassembly‐treated cells indicated the loss of mitochondrial membrane potential, implying the appearance of early apoptosis (Figure 4F). Therefore, we further studied the apoptosis of 4T1 cells. According to Figures 4G, H, the order of apoptosis rates of 4T1 cells was Taxol (57.5%) > PTX‐Cy‐Se NPs (49.3%) > PTX‐Cy‐S NPs (43.8%) > PTX‐Cy‐C NPs (28.5%). Among them, the apoptosis rate of PTX‐Cy‐Se NPs was closer to Taxol and consistent with cytotoxicity.

### 2.5 Pharmacokinetics and Biodistribution

**FIGURE 5:**
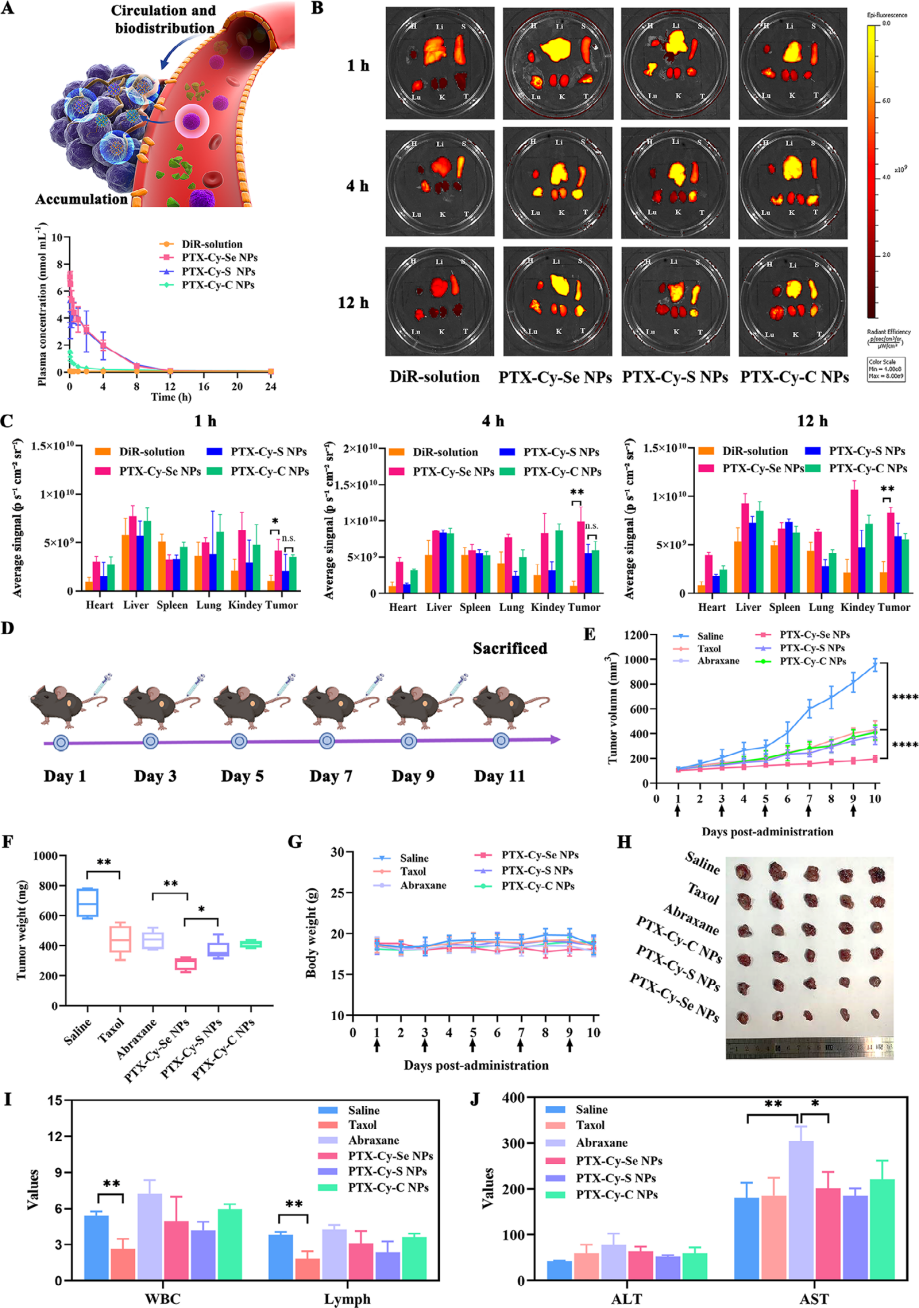
Pharmacokinetics, biodistribution, and antitumor effects. (A) Pharmacokinetic profiles of prodrug nanoassemblies within 24 h after administration (*n* = 3). (B–C) Fluorescence images and quantification of main organs and tumor after administration of DiR and DiR‐labeled prodrug nanoassemblies at various intervals (1 h, 4 h and 12 h, *n* = 3). (D) Diagram of the dosing cycle of 10 mg kg^−1^ of Taxol, Abraxane, PTX‐Cy‐Se NPs, PTX‐Cy‐S NPs, and PTX‐Cy‐C NPs for the treatment of Lewis tumors. (E) Tumor growth profiles (*n* = 5), (F) tumor weights (*n* = 5), (G) body weights (*n* = 5), (H) tumor photos (*n* = 5), (I) hematologic analysis (*n* = 3), and (J) hepatorenal function parameters (*n* = 3) of Lewis tumor‐bearing mice after five doses.

As intravenous agents, it is necessary to assess the hemocompatibility of the PTX‐Cy‐Se NPs, PTX‐Cy‐S NPs, and PTX‐Cy‐C NPs before conducting in vivo studies. Supplementary Figure S12 revealed that the hemolysis percentage of PTX‐Cy‐Se NPs, PTX‐Cy‐S NPs, and PTX‐Cy‐C NPs was well below 5%, which would not damage the erythrocytes and meet the requirements of intravenous injection. Then, the pharmacokinetics of the DiR‐labeled prodrug nanoassemblies were examined (Figure 5A and Supplementary Table S5). Free DiR was rapidly cleared with a low area under the curve (AUC). In contrast, the AUC of PTX‐Cy‐Se NPs, PTX‐Cy‐S NPs and PTX‐Cy‐C NPs was 11.3, 10.9, and 2.3 times higher than that of free DiR. PTX‐Cy‐S NPs, and PTX‐Cy‐Se NPs had better pharmacokinetics than PTX‐Cy‐C NPs, which should be attributed to their better assembly stability. Prodrug nanoassemblies with good assembly stability could maintain the integrity of the nanostructures to the maximum extent in plasma, slowing down the degradation of the prodrug nanoassemblies. Subsequently, the tumor accumulation and biodistribution of PTX‐Cy‐Se NPs, PTX‐Cy‐S NPs, and PTX‐Cy‐C NPs were investigated by 4T1 tumor‐bearing mice (Figures 5B, C). Consistent with the pharmacokinetic characteristics, free DiR was rapidly cleared in vivo, with significant accumulation only in the liver, spleen, and lung, while negligible accumulation was observed in tumor sites. However, prodrug nanoassemblies displayed pronounced tumor accumulation after the administration. PTX‐Cy‐Se NPs, with superior assembly stability, displayed enhanced tumor accumulation.

### 2.6 In Vivo Antitumor Effects

In the clinic, lung cancer and breast cancer were the two main therapeutic indications for PTX. Therefore, we evaluated the therapeutic effects of prodrug nanoassemblies on Lewis tumors and 4T1 tumors. As illustrated in Figures 5D–H, the untreated saline group showed rapid tumor growth. In contrast, Taxol and commercially available Abraxane had a certain effect on tumor suppression. PTX‐Cy‐S NPs and PTX‐Cy‐C NPs exhibited comparable antitumor effects compared to Abraxane. But PTX‐Cy‐Se NPs displayed the strongest tumor inhibition effect because PTX‐Cy‐Se NPs possessed higher tumor accumulation and rapid intracellular PTX release, which amplified the multiple pathway‐mediated apoptosis. H&E, TUNEL, and Ki‐67 staining of tumor tissues further confirmed that PTX‐Cy‐Se NPs could induce extensive apoptosis and inhibit tumor proliferation (Supplementary Figures S13–S14). Additionally, a significant reduction in the counts of white blood cells and lymphocytes was observed in mice of the Taxol group, revealing the occurrence of myelosuppression (Figure 5I). In addition, the aspartate aminotransferase (AST) level of mice in the Abraxane group was significantly elevated, indicating liver injury (Figure 5J). In contrast, mice in the prodrug nanoassemblies groups exhibited superior systemic safety (Supplementary Figures S15–S16).

**FIGURE 6:**
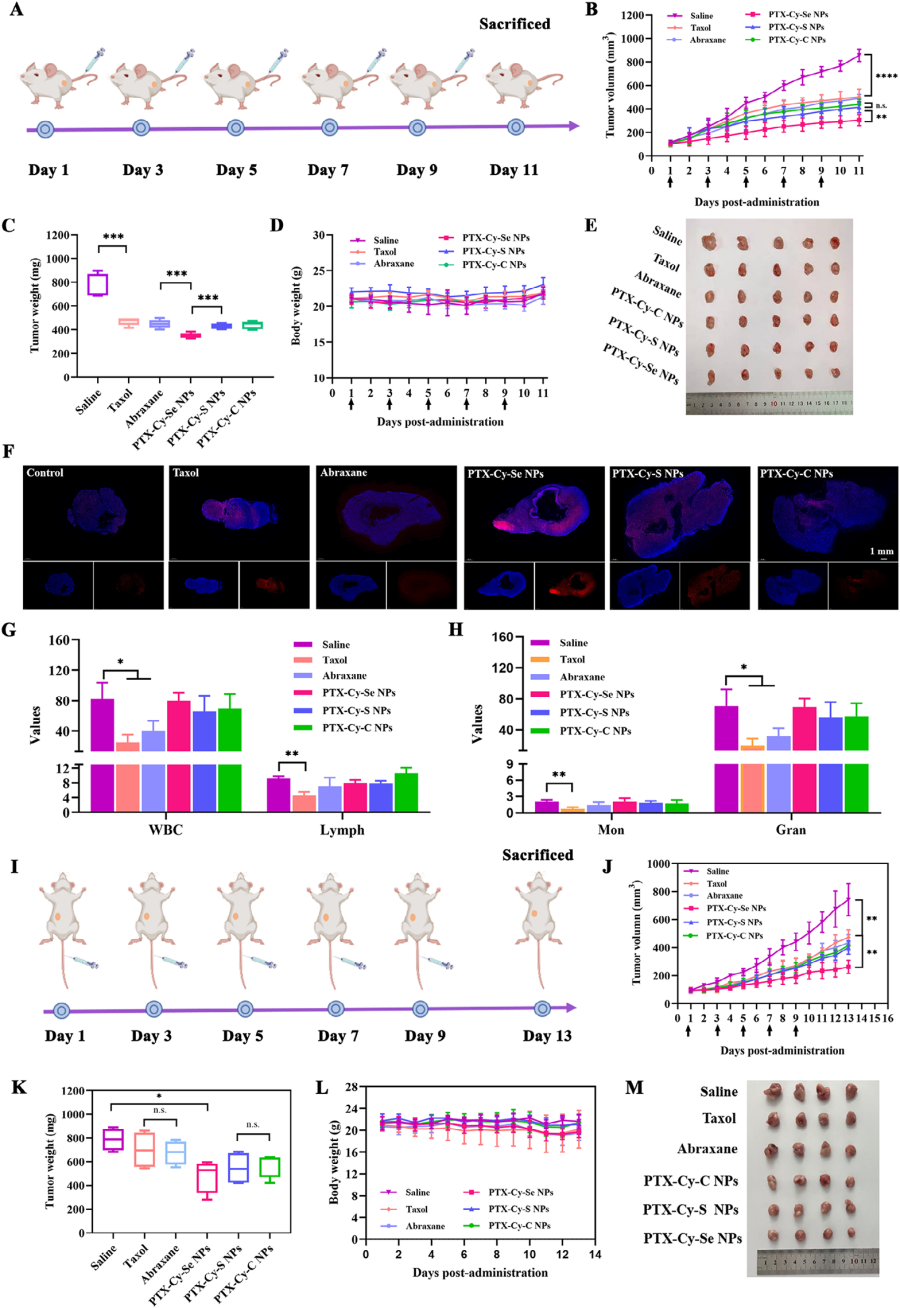
Antineoplastic therapy of heterotopic and orthotopic 4T1 tumors by prodrug nanoassemblies. (A) Diagram of the dosing cycle for heterotopic 4T1 tumor‐bearing mice. (B) Tumor growth profiles (*n* = 5), (C) tumor weights (*n* = 5), (D) body weights (*n* = 5), (E) tumor photos (*n* = 5), and (G–H) hematologic analysis (*n* = 3) of heterotopic 4T1 tumor‐bearing mice treated with 10 mg kg^−1^ of Taxol, Abraxane, PTX‐Cy‐Se NPs, PTX‐Cy‐S NPs and PTX‐Cy‐C NPs. (F) Intratumoral ROS stained with dihydroethidium (DHE). Scale bar: 1 mm. (I) Timeline of drug administration for the orthotopic 4T1 tumor model. (J) Tumor growth profiles (*n* = 4), (K) tumor weights (*n* = 4), (L) body weights (*n* = 4), and (M) tumor photos (*n* = 4) of orthotopic 4T1 tumor‐bearing mice after five doses.

4T1 cells are representative cells for the study of paclitaxel‐associated breast cancer treatment with high reproducibility and well‐established manipulation techniques. Therefore, 4T1 tumor cells were chosen in the antitumor studies. For heterotopic breast cancer, PTX‐Cy‐Se NPs also exhibited the best tumor suppression. Figures 6A–E revealed that the tumor volume and tumor weight of mice in the PTX‐Cy‐Se NPs group were the smallest. Staining of tumor tissue showed that PTX‐Cy‐Se NPs induced widespread apoptosis of tumor cells with more effective tumor inhibition (Supplementary Figures S17–S19). In addition, PTX‐Cy‐Se NPs intensely elevated ROS levels in tumor tissues, which promoted tumor oxidative stress (Figure 6F). Similarly, the hematologic analysis disclosed dramatic reductions in the counts of white blood cells, lymphocytes, monocytes, and neutrophils of the mice in the Taxol and Abraxane groups, revealing the myelosuppression and systemic toxicity (Figures 6G, H). In contrast, negligible changes in hematologic analysis and hepatorenal function were observed in prodrug nanoassemblies, indicating the excellent in vivo safety (Supplementary Figures S20–S21).

To further evaluate the therapeutic effect and anti‐metastatic ability of PTX‐Cy‐Se NPs, PTX‐Cy‐S NPs, and PTX‐Cy‐C NPs, the orthotopic breast cancer model was constructed. The antitumor efficacy of PTX‐Cy‐S NPs and PTX‐Cy‐C NPs was comparable to that of Abraxane and Taxol (Figures 6I–M). However, PTX‐Cy‐Se NPs still displayed the best antitumor efficacy. In addition, since the antitumor effects of the prodrug nanoassemblies were associated with oxidative stress, the expression of NOX4 in tumor sites was examined, as NOX4 was an enzyme closely associated with ROS generation. As shown in Supplementary Figure S22, the expression of NOX4 was elevated in the PTX‐Cy‐Se NPs group, which indicated that the treatment with PTX‐Cy‐Se NPs could aggravate the oxidative stress and promote tumor cell apoptosis. H&E staining showed that there was large‐scale apoptosis of tumor cells after treatment with PTX‐Cy‐Se NPs (Supplementary Figure S23). Ki‐67 and TUNEL staining of tumor tissues further verified that PTX‐Cy‐Se NPs could induce more apoptosis and inhibit the proliferation of tumor cells (Supplementary Figures S24–S25). In addition, CD31 staining showed that the prodrug nanoassemblies, especially PTX‐Cy‐Se NPs, could reduce tumor angiogenesis after treatment (Supplementary Figure S26). Moreover, PTX‐Cy‐Se NPs could significantly inhibit the lung metastasis of tumors compared with PTX‐Cy‐S NPs and PTX‐Cy‐C NPs (Supplementary Figure S27). Additionally, the hematologic analysis suggested that the prodrug nanoassemblies displayed better safety compared to Taxol (Supplementary Figures S28–S29).

Overall, the reasons for the more effective antitumor efficacy of PTX‐Cy‐Se NPs were as follows: firstly, PTX‐Cy‐Se NPs displayed higher tumor accumulation than PTX‐Cy‐S NPs and PTX‐Cy‐C NPs. Secondly, PTX‐Cy‐Se NPs could release more PTX in tumor cells. Finally, PTX‐Cy‐Se NPs induced more ROS generation and significantly inhibited the GSH/GPx antioxidant system, which aggravated tumor oxidative stress and promoted tumor cell apoptosis.

## 3 Conclusions

Redox‐responsive nanomedicines, as a precise and intelligent tumor treatment scheme, realized on‐command drug delivery to tumor sites. Compared to conventional redox‐responsive prodrugs, the integration of the response module and modification module in a five‐membered ring significantly reduced the complexity of the prodrugs, which minimized the use of non‐pharmacological moieties from the source of structure design. Surprisingly, the five‐membered ring structure facilitated self‐assembly due to the stronger chalcogen bonding interactions and the nearly 90° bond angle, especially for PTX‐Cy‐Se. In addition, we investigated the redox activation of prodrug nanoassemblies, especially the differences in the redox response mechanisms of five‐membered cyclic chalcogen bonds. Importantly, the prodrug nanoassemblies induced tumor cell apoptosis through multiple pathways, including preventing microtubule protein depolymerization, blocking the GSH/GPx antioxidant system, instigating ROS overproduction, and disrupting the mitochondrial membrane potential. Among them, PTX‐Cy‐Se NPs displayed potent antitumor efficiency, which was attributed to the superior self‐assembly stability, strong redox responsiveness, and systematic modulation of tumor redox homeostasis. Our studies broke through the bottlenecks of modular design of prodrug nanoassemblies and provided an insightful direction for effective tumor therapy.

## Author Contributions

**Yixin Sun**: methodology, experiment, visualization, and writing. **Shiyi Zuo**: experiment. **Wenfeng Zang**: experiment. **Lingxiao Li**: analytics. **Xianbao Shi**: analytics. **Mingzhong Li**: visualization and writing. **Zhonggui He**: conceptualization and writing. **Bingjun Sun**: conceptualization and writing. **Jin Sun**: conceptualization and writing.

## Conflicts of Interest

The authors declare no conflicts of interest.

## Supporting information




**Supporting File 1**: exp270152‐sup‐0001‐SuppMat.docx.

## Data Availability

The data that support the findings of this study are available from the corresponding author upon reasonable request.

## References

[1] G. Bocci and R. S. Kerbel , “Pharmacokinetics of Metronomic Chemotherapy: A Neglected but Crucial Aspect,” Nature Reviews Clinical Oncology 13 (2016): 659–673, 10.1038/nrclinonc.2016.64. 27184418

[2] B. A. Baldo and N. H. Pham , “Adverse Reactions to Targeted and Non‐Targeted Chemotherapeutic Drugs with Emphasis on Hypersensitivity Responses and the Invasive Metastatic Switch,” Cancer and Metastasis Reviews 32 (2013): 723–761, 10.1007/s10555-013-9447-3. 24043487 PMC7102343

[3] C. Ding , C. Chen , X. Zeng , H. Chen , and Y. Zhao , “Emerging Strategies in Stimuli‐Responsive Prodrug Nanosystems for Cancer Therapy,” ACS Nano 16 (2022): 13513–13553, 10.1021/acsnano.2c05379. 36048467

[4] Z. Shi , Q. Song , R. Gostl , and A. Herrmann , “Mechanochemical Activation of Disulfide‐Based Multifunctional Polymers for Theranostic Drug Release,” Chemical Science 12 (2020): 1668–1674, 10.1039/D0SC06054B. 34163927 PMC8179261

[5] F. Gong , N. Yang , X. Wang , et al., “Tumor Microenvironment‐Responsive Intelligent Nanoplatforms for Cancer Theranostics,” Nano Today 32 (2020): 100851, 10.1016/j.nantod.2020.100851.

[6] Q. Mou , Y. Ma , X. Zhu , and D. Yan , “A Small Molecule Nanodrug Consisting of Amphiphilic Targeting Ligand‐Chemotherapy Drug Conjugate for Targeted Cancer Therapy,” Journal Control Release 230 (2016): 34–44, 10.1016/j.jconrel.2016.03.037. 27040815

[7] C. Song , Y. Li , T. Li , et al., “Long‐Circulating Drug‐Dye‐Based Micelles With Ultrahigh pH‐Sensitivity for Deep Tumor Penetration and Superior Chemo‐Photothermal Therapy,” Advanced Functional Materials 30 (2020): 1906309, 10.1002/adfm.201906309.

[8] E. C. Cheung and K. H. Vousden , “The Role of ROS in Tumour Development and Progression,” Nature Reviews Cancer 22 (2022): 280–297, 10.1038/s41568-021-00435-0. 35102280

[9] X. Zhang , L. Han , M. Liu , et al., “Recent Progress and Advances in Redox‐Responsive Polymers as Controlled Delivery Nanoplatforms,” Materials Chemistry Frontiers 1 (2017): 807–822, 10.1039/C6QM00135A.

[10] Y. Tian , R. Guo , Y. Jiao , et al., “Redox Stimuli‐Responsive Hollow Mesoporous Silica Nanocarriers for Targeted Drug Delivery in Cancer Therapy,” Nanoscale Horizons 1 (2016): 480–487, 10.1039/C6NH00139D. 32260712

[11] F. Sun , Q. Zhu , T. Li , et al., “Regulating Glucose Metabolism With Prodrug Nanoparticles for Promoting Photoimmunotherapy of Pancreatic Cancer,” Advanced Science (Weinh) 8 (2021): 2002746, 10.1002/advs.202002746. PMC7887571 33643795

[12] J. Ye , B. Hou , F. Chen , et al., “Bispecific Prodrug Nanoparticles Circumventing Multiple Immune Resistance Mechanisms for Promoting Cancer Immunotherapy,” Acta Pharmaceutica Sinica B 12 (2022): 2695–2709, 10.1016/j.apsb.2021.09.021. 35755274 PMC9214055

[13] D. Trachootham , J. Alexandre , and P. Huang , “Targeting Cancer Cells by ROS‐Mediated Mechanisms: A Radical Therapeutic Approach?,” Nature Reviews Drug Discovery 8 (2009): 579–591, 10.1038/nrd2803. 19478820

[14] Y. Liu , S. Zhai , X. Jiang , et al., “Intracellular Mutual Promotion of Redox Homeostasis Regulation and Iron Metabolism Disruption for Enduring Chemodynamic Therapy,” Advanced Functional Materials 31 (2021): 2010390, 10.1002/adfm.202010390.

[15] D. Jana and Y. Zhao , “Strategies for Enhancing Cancer Chemodynamic Therapy Performance,” Exploration 2 (2022): 20210238, 10.1002/EXP.20210238. 37323881 PMC10191001

[16] S. Saikolappan , B. Kumar , G. Shishodia , S. Koul , and H. K. Koul , “Reactive Oxygen Species and Cancer: A Complex Interaction,” Cancer Letters 452 (2019): 132–143, 10.1016/j.canlet.2019.03.020. 30905813

[17] L. Cao , J. Zhang , Y. Du , et al., “Selenite Induced Breast Cancer MCF7 Cells Apoptosis Through Endoplasmic Reticulum Stress and Oxidative Stress Pathway,” Chemico‐Biological Interactions 349 (2021): 109651, 10.1016/j.cbi.2021.109651. 34520753

[18] G. Chen , Y. Yang , Q. Xu , et al., “Self‐Amplification of Tumor Oxidative Stress With Degradable Metallic Complexes for Synergistic Cascade Tumor Therapy,” Nano Letters 20 (2020): 8141–8150, 10.1021/acs.nanolett.0c03127. 33172280

[19] C. R. Corso and A. Acco , “Glutathione System in Animal Model of Solid Tumors: From Regulation to Therapeutic Target,” Critical Reviews in Oncology/Hematology 128 (2018): 43–57, 10.1016/j.critrevonc.2018.05.014. 29958630

[20] B. Sun , C. Luo , X. Zhang , et al., “Probing the Impact of Sulfur/Selenium/Carbon Linkages on Prodrug Nanoassemblies for Cancer Therapy,” Nature Communications 10 (2019): 3211, 10.1038/s41467-019-11193-x. PMC6642185 31324811

[21] Y. Yang , S. Zuo , J. Zhang , et al., “Prodrug Nanoassemblies Bridged by Mono‐/Di‐/Tri‐Sulfide Bonds: Exploration Is for Going Further,” Nano Today 44 (2022): 101480, 10.1016/j.nantod.2022.101480.

[22] B. Sun , C. Luo , W. Cui , J. Sun , and Z. He , “Chemotherapy Agent‐Unsaturated Fatty Acid Prodrugs and Prodrug‐Nanoplatforms for Cancer Chemotherapy,” Journal Control Release 264 (2017): 145–159, 10.1016/j.jconrel.2017.08.034. 28844757

[23] B. Sun , C. Luo , H. Yu , et al., “Disulfide Bond‐Driven Oxidation‐ and Reduction‐Responsive Prodrug Nanoassemblies for Cancer Therapy,” Nano Letters 18 (2018): 3643–3650, 10.1021/acs.nanolett.8b00737. 29726685

[24] D. Abegg , G. Gasparini , D. G. Hoch , et al., “Strained Cyclic Disulfides Enable Cellular Uptake by Reacting With the Transferrin Receptor,” Journal of the American Chemical Society 139 (2017): 231–238, 10.1021/jacs.6b09643. 28001050

[25] E. Bartolami , D. Basagiannis , L. Zong , et al., “Diselenolane‐Mediated Cellular Uptake: Efficient Cytosolic Delivery of Probes, Peptides, Proteins, Artificial Metalloenzymes and Protein‐Coated Quantum Dots,” Chemistry (Weinheim An Der Bergstrasse, Germany) 25 (2019): 4047–4051, 10.1002/chem.201805900. 30815941

[26] X. Li , B. Zhang , C. Yan , et al., “A Fast and Specific Fluorescent Probe for Thioredoxin Reductase That Works via Disulphide Bond Cleavage,” Nature Communications 10 (2019): 2745, 10.1038/s41467-019-10807-8. PMC6588570 31227705

[27] X. Li , Y. Hou , J. Zhao , J. Li , S. Wang , and J. Fang , “Combination of Chemotherapy and Oxidative Stress to Enhance Cancer Cell Apoptosis,” Chemical Science 11 (2020): 3215–3222, 10.1039/C9SC05997K. 34122827 PMC8157308

[28] L. Vogel , P. Wonner , and S. M. Huber , “Chalcogen Bonding: An Overview,” Angewandte Chemie International Edition 58 (2019): 1880–1891, 10.1002/anie.201809432. 30225899

[29] W. Hou and H. Xu , “Incorporating Selenium into Heterocycles and Natural Products Horizontal Line From Chemical Properties to Pharmacological Activities,” Journal of Medicinal Chemistry 65 (2022): 4436–4456, 10.1021/acs.jmedchem.1c01859. 35244394

[30] Y. Yang , B. Sun , S. Zuo , et al., “Trisulfide Bond–Mediated Doxorubicin Dimeric Prodrug Nanoassemblies with High Drug Loading, High Self‐Assembly Stability, and High Tumor Selectivity,” Science Advances 6: eabc1725, 10.1126/sciadv.abc1725. PMC7673695 33148644

[31] Q. Pei , X. Hu , S. Liu , Y. Li , Z. Xie , and X. Jing , “Paclitaxel Dimers Assembling Nanomedicines for Treatment of Cervix Carcinoma,” Journal Control Release 254 (2017): 23–33, 10.1016/j.jconrel.2017.03.391. 28359677

[32] T. Lu , Molclus Program, Version 1.9 . 2020, http://www.keinsci.com/research/molclus.html.

[33] S. Grimme , C. Bannwarth , and P. Shushkov , “A Robust and Accurate Tight‐Binding Quantum Chemical Method for Structures, Vibrational Frequencies, and Noncovalent Interactions of Large Molecular Systems Parameterized for all Spd‐Block Elements (Z = 1–86),” Journal of Chemical Theory and Computation 13 (2017): 1989–2009, 10.1021/acs.jctc.7b00118. 28418654

[34] Y. N. Bai , X. N. Wang , Y. Z. Lu , et al., “Microbial Selenite Reduction Coupled to Anaerobic Oxidation of Methane,” Science of the Total Environment 669 (2019): 168–174, 10.1016/j.scitotenv.2019.03.119. 30878925

