# Supplementary material for: Modification‐Response Integrated Modules Driven Cyclization‐Dependent Prodrug Self‐Assembly for Reshaping Pro‐Apoptotic Tumor Redox Homeostasis
Source: Exploration (Beijing). 2026 Mar 10;6(2):20240448. doi: 10.1002/EXP.20240448 (PMC13094520; doi:10.1002/EXP.20240448)
Supplement: Supplementary file 1 — Supporting File 1: exp270152‐sup‐0001‐SuppMat.docx. [file EXP2-6-20240448-s001.docx]

**Supporting Information for**

**Modification-response integrated modules driven cyclization-dependent prodrug self-assembly for reshaping pro-apoptotic tumor redox homeostasis**

Authors: Yixin Sun^1^, Shiyi Zuo^1^, Wenfeng Zang^1^, Lingxiao Li^1^, Xianbao Shi^3^, Mingzhong Li^4^, Zhonggui He^1, 2, *^, Bingjun Sun^1, 2, *^, Jin Sun^1, 2, *^

Affiliations:

^1^Department of Pharmaceutics, Wuya College of Innovation, Shenyang Pharmaceutical University, Shenyang, China

^2^Joint International Research Laboratory of Intelligent Drug Delivery Systems, Ministry of Education, Shenyang Pharmaceutical University, Shenyang, China

^3^Department of Pharmacy, The First Affiliated Hospital of Jinzhou Medical University, Jinzhou, China

^4^Leicester School of Pharmacy, De Montfort University, Leicester, UK

*Corresponding authors

Jin Sun, Bingjun Sun, Zhonggui He,

Department of Pharmaceutics, Wuya College of Innovation, Shenyang Pharmaceutical University, Shenyang, China

E-mail address: sunjin@syphu.edu.cn; sunbingjun_spy@sina.com; hezhgui_student@aliyun.com

**Supplemental materials and methods**

**Materials.** Paclitaxel, bicinchoninic acid (BCA) protein assay kit and dithiothreitol (DTT) were bought from Dalian Meilun Biotechnology Co., Ltd. 3-Bromo-2-(bromomethyl) propionic acid and selenium powder were purchased from Shanghai Aladdin Biochemical Technology Co., Ltd. Tubulin-Tracker Red and mitochondrial membrane potential assay kit with JC-1 were bought from Beyotime Biotechnology. 1,2-distearoyl-sn-glycero-3-phosphoethanolamine-N [methoxy(polyethyleneglycol)-2000] (DSPE-mPEG_2000_) was bought from AVT (Shanghai) Pharmaceutical Tech Co., Ltd. Coumarin-6 (C6), Glutathione Peroxidase (GPx) and Glutathione Reductases (GR) activity assay kit and Annexin V-FITC/PI apoptosis assay kit were purchased from Beijing Solarbio Science and Technology Co., Ltd. 24-well plates were sourced from Wuxi NEST Biotechnology Co., Ltd.

**Synthesis of 1,2-diselenolane-4-carboxylic acid.** Selenium powder (1.58 g, 20 mmol) and 30 mL of absolute ethanol were placed in a 250 mL round-bottom three-necked flask, and sodium borohydride (1.51 g, 40 mmol) was solubilized in 40 mL of absolute ethanol. The sodium borohydride solution was slowly dripped into the above solution and stirred for 0.5 h at 0°C. Later, the second portion of selenium powder (1.97 g, 25 mmol) was poured into the flask and reacted at 80°C for 0.5 h. Under nitrogen protection, 3-bromo-2-(bromomethyl) propionic acid (4.92 g, 20 mmol) was added dropwise with homogeneous stirring, and the reaction was allowed to proceed overnight at 25°C. Finally, filtration and dilution with a large amount of water, multiple liquid-liquid extractions with ethyl acetate were performed to collect the target product. The dark red-brown solid was obtained after drying with anhydrous magnesium sulfate and evaporation under reduced pressure.

**Synthesis of PTX-Cy-Se, PTX-Cy-S and PTX-Cy-C prodrugs.** Dichloromethane was used to dissolve PTX (341.57 mg, 0.4 mmol), 1,2-diselenolane-4-carboxylic acid or 1,2-dithiolane-4-carboxylic acid or cyclopentanecarboxylic acid (0.4 mmol), EDCI (191.7 mg, 1 mmol) and DMAP (9.78 mg, 0.08 mmol) and stirred for 12 h (25°C). Dichloromethane was removed by rotary evaporation, and then reconstituted with acetonitrile and purified by preparative liquid chromatography. The final products were characterized by HRMS, ^1^H NMR and HPLC. The yields of prodrugs were approximately 35% (PTX-Cy-Se), 45% (PTX-Cy-S) and 48% (PTX-Cy-C).

**Construction and characterization of prodrug nanoassemblies.** After dissolving PTX-Cy-Se, PTX-Cy-S or PTX-Cy-C prodrugs (4 mg) and DSPE-mPEG_2000_ (1 mg) in anhydrous ethanol, the drug-containing solution was injected into deionized water (4 mL) with stirring to obtain the prodrug nanoassemblies (PTX-Cy-Se NPs, PTX-Cy-S NPs and PTX-Cy-C NPs). The non-PEGylated prodrug nanoassemblies were fabricated without DSPE-mPEG_2000_. The prodrugs, DiR/C6 and DSPE-mPEG_2000_ were dissolved in anhydrous ethanol to yield DiR/C6-labeled prodrug nanoassemblies. The Nano ZS Zetasizer instrument and transmission electron microscopy were employed to characterize particle size, zeta potential and morphology. The particle size of PTX-Cy-Se NPs, PTX-Cy-S NPs, and PTX-Cy-C NPs was determined when stored at 4°C (30 days) and room temperature (7 days). Furthermore, particle size changes of PTX-Cy-Se NPs, PTX-Cy-S NPs, and PTX-Cy-C NPs were determined after co-incubation with a more demanding medium (PBS containing 10% FBS) at 37°C.

**Assembly mechanism.** The structures of prodrugs were optimized and the bond angles of -C-Se-Se-C, -C-S-S-C and -C-C-C-C in prodrugs were calculated using Gaussian. The molecular dynamics simulations were carried out using Gromacs 2019. Intermolecular forces, including π-π stacking, hydrophobic forces, hydrogen bonding and chalcogen bonding, were resolved by Discovery Studio 4.5 software. Besides, the presence of hydrogen bonding and hydrophobic forces was investigated by co-incubation of PTX-Cy-Se NPs, PTX-Cy-S NPs, and PTX-Cy-C NPs with urea, sodium dodecyl sulfate (SDS) and Triton X-100.

**Redox dual-response activation.** PTX-Cy-Se NPs, PTX-Cy-S NPs, and PTX-Cy-C NPs were homogeneously blended in PBS (pH: 7.4; 1% SDS and 10% ethanol) containing H_2_O_2_ (0.1 mM, 1 mM and 2 mM) or DTT (1 mM, 2 mM and 5 mM) at 37°C. Samples were taken at scheduled intervals and analyzed by HPLC. To explore the activation mechanism, prodrug nanoassemblies were incubated with PBS containing 1 mM H_2_O_2_ or DTT for 2 h, and the intermediates were confirmed by HRMS, XPS and ^1^H NMR.

**Cellular uptake.** 4T1, A549 and 3T3 cells were cultivated in 12-well plates (2 × 10^5^ cells well^-1^). Taxol, PTX-Cy-Se NPs, PTX-Cy-S NPs, and PTX-Cy-C NPs (10 μM, PTX equivalent) acted on the cells for another 6 h and 12 h. Subsequently, cells were washed, collected and fragmented by ultrasonication. After protein precipitation, the intracellular PTX concentration was quantified by HPLC. The protein concentration in the sample was quantified by making a standard curve using the BCA protein assay kit.

**Cytotoxicity.** Tumor cells (4T1 cells, A549 cells and B16-F10 cells: 2 × 10^3^ cells/well) and normal cells (3T3 cells) were cultivated in 96-well plates. Subsequently, incubation was continued for 48 h with Taxol, PTX-Cy-Se NPs, PTX-Cy-S NPs, and PTX-Cy-C NPs. After 4 h incubation with MTT, the formazan in live cells was solubilized with DMSO, and the absorbance of the well plates was tested at 490 nm or 570 nm with a microplate reader (BioTek, USA). The half-maximal inhibitory concentration (IC_50_) was gained by nonlinear fitting in GraphPad Prism 8 with molar concentration as the X-axis and cell viability as the Y-axis.

**Microtubule polymerization assay.** 4T1 cells (1 × 10^5^ cells well^-1^) were cultured on coverslips in 24-well plates. Taxol and three prodrug nanoassemblies (100 nM, PTX equivalent) were used to treat the cells for another 48 h. After culture, 4% paraformaldehyde was utilized to incubate the cells. Then, the cells were stained with DAPI for 10 min and treated with Tubulin-Tracker Red fluorescent probes. Finally, the fluorescence of microtubules and nuclei was visualized by CLSM.

**Detection of intracellular ROS.** 4T1 cells (1 × 10^5^ cells well^-1^) were cultured on coverslips in 24-well plates. Later, Taxol and three prodrug nanoassemblies (100 nM, PTX equivalent) were used to treat the cells for 12 h. Next, cells were rinsed and loaded with DCFH-DA. After washing off the excess probes, the coverslips were observed using CLSM. For fluorescence quantification, drug-treated cells needed to be washed and loaded with DCFH-DA probes. Afterward, cells were rinsed, digested by trypsin (0.25%, w/w), terminated digestion, centrifuged at 4°C (1500 rpm, 5 min), and resuspended in PBS. The green fluorescence was detected with the BD FACS Celesta flow cytometry (Becton, Dickinson and Company, USA).

**Blocking of the GSH/GPx antioxidant system.** The GSH/GPx antioxidant system mainly consisted of three important components, namely GSH, GPx and GR. For GSH detection, 4T1 cells (2 × 10^5^ cells well^-1^) were planted in 6-well plates. After 12 h of incubation, Taxol and three prodrug nanoassemblies (100 nM, PTX equivalent) were used to treat the cells. Next, cells were rinsed, digested, and centrifuged at 4°C (3000 rpm, 5 min) and resuspended in 200 μL of PBS. The collected cells were quantified with the GSH and GSSG assay kit.

For the activity analysis of GPx and GR, Taxol, PTX-Cy-Se NPs, PTX-Cy-S NPs, and PTX-Cy-C NPs (100 nM, PTX equivalent) were administered to 4T1 cells for 12 h. After incubation, cells were processed by the Glutathione Peroxidase activity assay kit and the Glutathione Reductases (GR) activity assay kit. The inhibition of GPx and GR was calculated according to the instructions.

**Detection of mitochondrial membrane potential.** Taxol, PTX-Cy-Se NPs, PTX-Cy-S NPs, and PTX-Cy-C NPs (100 nM, PTX equivalent) were administered to 4T1 cells for 12 h. Later, cells were processed as described in the operating procedure of the mitochondrial membrane potential assay kit with JC-1, and the fluorescence was observed through CLSM.

**Apoptosis assay.** 4T1 cells were grown on coverslips in 24-well plates (1 × 10^5^ cells well^-1^) and 12-well plates (2 × 10^5^ cells well^-1^), and the cells were incubated with Taxol, PTX-Cy-Se NPs, PTX-Cy-S NPs, and PTX-Cy-C NPs (100 nM, PTX equivalent) for 12 h. For CLSM analysis, 4% paraformaldehyde was utilized to incubate the cells, and DAPI stained for nuclei. For fluorescence quantification, the cells needed to be washed and processed following the protocol of the Annexin V-FITC apoptosis detection kit.

***In vitro* hemocompatibility.** Fresh blood from healthy rats was taken, centrifuged to collect blood cells, cleaned with saline, and configured into 2% erythrocyte suspension. PTX-Cy-Se NPs, PTX-Cy-S NPs, and PTX-Cy-C NPs, Triton X-100 and saline were blended with the erythrocyte suspension. Triton X-100 and saline were employed as controls. Subsequently, the cell suspension was allowed to incubate in an oscillator for 2 h (37°C). After centrifugation at 3000 rpm for 10 min, photographs were taken and the supernatant was aspirated to determine the absorbance values. Hemolysis percentage (HP%) = (A_Sample_-A_Saline_) / (A_Triton X-100_-A_Saline_) ×100%.

***In vivo* pharmacokinetics.** All animal studies were authorized by the Institutional Animal Ethical Care Committee (IAEC) of Shenyang Pharmaceutical University (SYXK 2020-0009, 17066) in strict compliance with the Guide for the Care and Use of Laboratory Animals. Sprague-Dawley rats (180-220 g) were utilized for pharmacokinetic evaluation. Free DiR and DiR-labeled PTX-Cy-Se NPs, PTX-Cy-S NPs, and PTX-Cy-C NPs (1 mg kg^-1^, DiR equivalent) were administered via intravenous injection. Whole blood from rats was collected and centrifuged (13000 rpm, 5 min) to obtain plasma samples. The rat plasma was kept at -80°C. After protein precipitation, DiR was determined by Varioskan Flash multimode microreader (Thermo Scientific, USA).

**Biodistribution.** 100 μL of suspension containing 5 × 10^6^ of 4T1 cells was injected percutaneously into the right posterior dorsum of dehairing BALB/c mice (n = 3). About 14 days later, free DiR and DiR-labeled PTX-Cy-Se NPs, PTX-Cy-S NPs, and PTX-Cy-C NPs (1 mg kg^-1^, DiR equivalent) were administered by intravenous injection when the tumor reached 300-400 mm^3^. After that, the main organs and the tumor were isolated at 1, 4, and 12 h. A noninvasive optical *in vivo* imaging system was applied to photograph and quantify the fluorescence intensity of the tissues.

***In vivo* antitumor effects.** Lung cancer and breast cancer models were selected according to the clinical indications of PTX. For the lung cancer model, 100 μL of suspension containing 5 × 10^6^ of Lewis lung cells was injected percutaneously into the right posterior dorsum of dehairing C57BL/6 mice (n = 5). Taxol, Abraxane, PTX-Cy-Se NPs, PTX-Cy-S NPs, and PTX-Cy-C NPs (10 mg kg^-1^, PTX equivalent) were administered on days 1, 3, 5, 7 and 9 when the tumor grew to about 100 mm^3^ (Tumor volume (mm^3^) = (Length × Width × Width) / 2). After five administrations, the main organs and the tumor were isolated for H&E pathological staining. Tumor tissues were stained for TUNEL and Ki-67 staining. In addition, hematologic and hepatorenal function analysis were performed.

BALB/c mice (n = 5) were used to establish the heterotopic breast cancer model by subcutaneous inoculation of 4T1 cells (5 × 10^6^) on the right hind back. Taxol, Abraxane, PTX-Cy-Se NPs, PTX-Cy-S NPs, and PTX-Cy-C NPs (10 mg kg^-1^, PTX equivalent) were administered by intravenous injection. Mice were sacrificed and subjected to a series of pathological and medical analyses as described above after a total of 5 doses administered on days 1, 3, 5, 7 and 9. For intratumoral ROS staining experiments, Taxol, Abraxane, PTX-Cy-Se NPs, PTX-Cy-S NPs, and PTX-Cy-C NPs (10 mg kg^-1^, PTX equivalent) were administered by intravenous injection. After 24 h of administration, dihydroethidium was stained in the tumor tissue. For the orthotopic breast cancer model, the suspension containing 1 × 10^6^ of 4T1 cells was injected into the mammary fat pad of female BALB/c mice (n = 4). The same dosage and cycle of administration as in the heterotopic 4T1 tumor model were adopted. After five cycles of treatment, mice were sacrificed and subjected to a series of pathological and medical analyses as described above. Moreover, immunofluorescence assays were performed for NOX4 enzymes, which were associated with ROS generation. In addition, CD31 staining was used to observe the vascular distribution of tumor tissues. Bouin 's solution was used to fix the lung, and the number of tumors on the surface of pulmonary lobes was recorded in detail.

**Statistics.** All quantitative data were presented in the form of means (SD). Statistical analysis was conducted using Student's t-test (two-tailed), and significance was denoted by n.s (no significance), P > 0.05, *P < 0.05, **P < 0.01, ***P < 0.001, and ****P < 0.0001.

**Supplementary Figures**


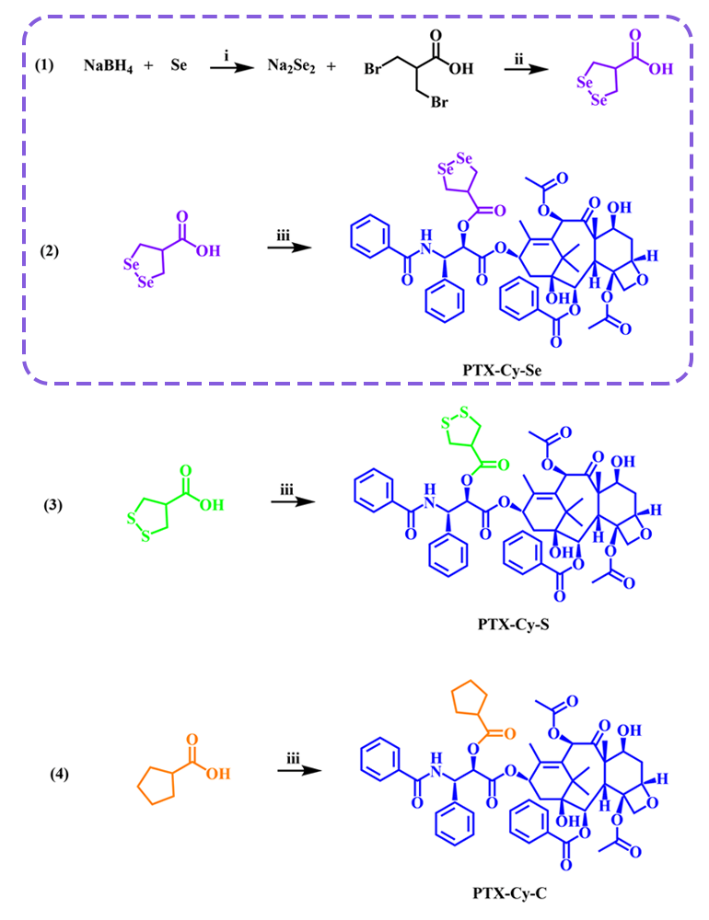


**Figure S****1.** Synthesis routes of prodrugs. (i) Ethanol, 0°C, 40 min; 80°C, 30 min; (ii) 25°C, 12 h; (iii) PTX, EDCI, DMAP, 25°C, 12 h.

**
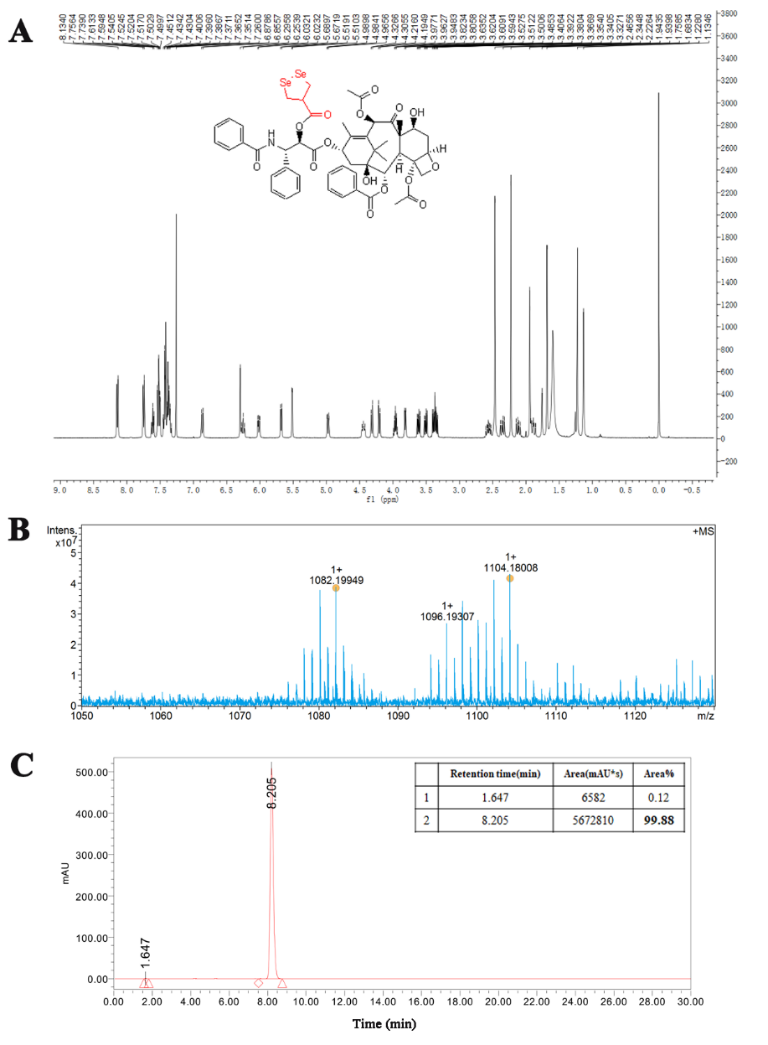
**

**Figure S2.** Characterizations of PTX-Cy-Se. (A) ^1^H NMR spectrum. (B) Mass spectrum. (C) The purity.

^1^H NMR (400 MHz, Chloroform-d) δ 8.14 (d, 2H, o-2O-ArH), 7.75 (d, 2H, o-3’NH-ArH), 7.64 - 7.57 (m, 1H, p-2O-ArH), 7.55 - 7.31 (m, 10H, 3’-ArH, 2O-ArH, 3’NH-ArH), 6.87 (d, 1H, 3’-NH-), 6.30 (s, 1H, 10-H), 6.25 (t, 1H, 13-H), 6.02 (dd, 1H, 3’-H), 5.68 (d, 1H, 2-H), 5.51 (d, 1H, 2’-H), 4.97 (dd, 1H, 5-H), 4.44 (t, 1H, 7-H), 4.32 (d, 1H, 20-HH), 4.21 (d, 1H, 20-HH), 3.96 (p, 1H, OOCCHCH_2_), 3.81 (d, 1H, 3-H), 3.61 (dd, 1H, OOCCHCH_2_), 3.51 (dd, 1H, OOCCHCH_2_), 3.37 (ddd, 2H, OOCCHCH_2_), 2.56 - 2.53 (m, 1H, 6-HH), 2.47 (s, 4H, 7-OH, 4O-Ac-H), 2.35 (dd, 1H, 14-H), 2.23 (s, 3H, 10O-Ac-H), 2.12 (dd, 1H, 14-H), 1.94 (s, 3H, 18-CH_3_), 1.93 - 1.85 (m, 1H, 6-HH), 1.76 (s, 1H, 1-OH), 1.68 (s, 3H, 19-CH_3_), 1.23 (s, 3H, 17-CH_3_), 1.13 (s, 3H, 16-CH_3_).

MS (ESI) m/z for C_51_H_56_NO_15_Se_2_ [M+H]^+^: 1082.19949.


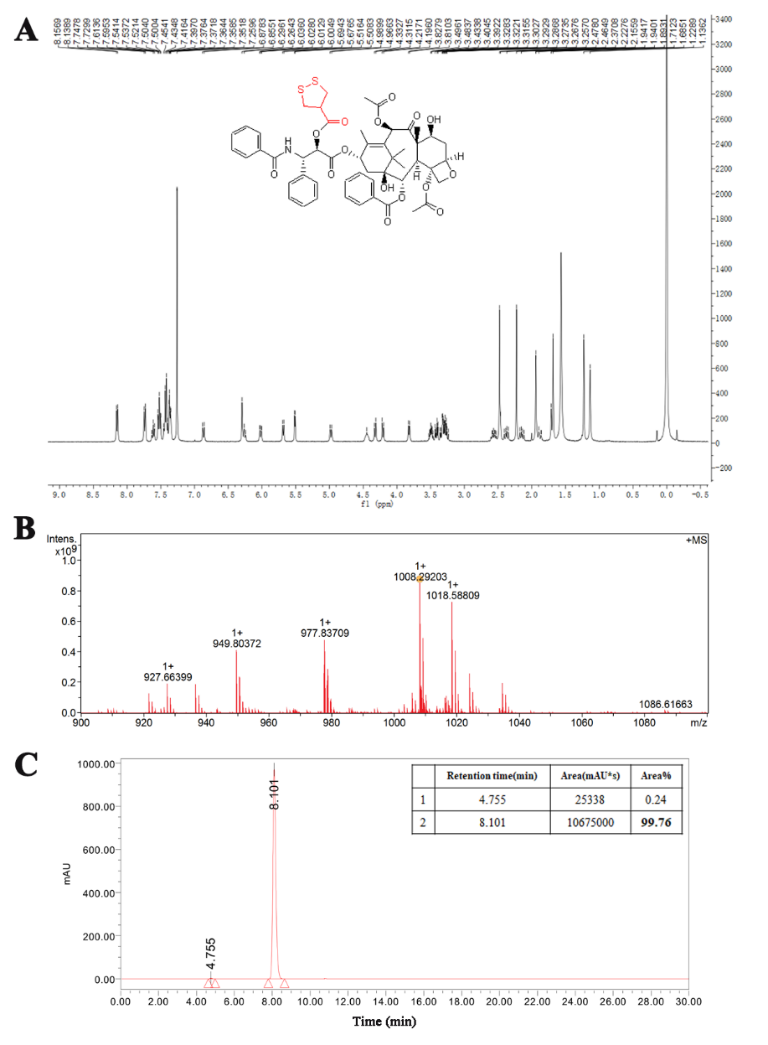


**Figure S3.** Characterizations of PTX-Cy-S. (A) ^1^H NMR spectrum. (B) Mass spectrum. (C) The purity.

^1^H NMR (400 MHz, Chloroform-d) δ 8.15 (d, 2H, o-2O-ArH), 7.74 (d, 2H, o-3’NH-ArH), 7.64 - 7.57 (m, 1H, p-2O-ArH), 7.57 - 7.30 (m, 10H, 3’-ArH and 2O-ArH and 3’NH-ArH), 6.87 (d, 1H, 3’-NH-), 6.30 (s, 1H, 10-H), 6.25 (d, 1H, 13-H), 6.02 (dd, 1H, 3’-H), 5.69 (d, 1H, 2-H), 4.98 (d, 1H, 5-H), 4.45 (t, 1H, 7-H), 4.32 (d, 1H, 20-HH), 4.21 (d, 1H, 20-HH), 3.82 (d, 1H, 3-H), 3.52-3.48 (m, 1H, OOCCHCH_2_), 3.41 (m, 1H, OOCCHCH_2_), 3.37 - 3.22 (m, 3H, OOCCHCH_2_, OOCCHCH_2_), 2.57 - 2.55 (m, 1H, 6-HH), 2.48 (s, 4H, 7-OH, 4O-Ac-H), 2.38 (dd, 1H, 14-H), 2.23 (s, 3H, 10O-Ac-H), 2.15 (dd, 1H, 14-H), 1.97 - 1.84 (m, 4H, 18-CH_3_, 6-HH), 1.71 (s, 1H, 1-OH), 1.69 (s, 3H, 19-CH_3_), 1.23 (s, 3H, 17-CH_3_), 1.14 (s, 3H, 16-CH_3_).

MS (ESI) m/z for C_51_H_55_NO_15_S_2_Na [M+Na]^+^: 1008.29203.

**
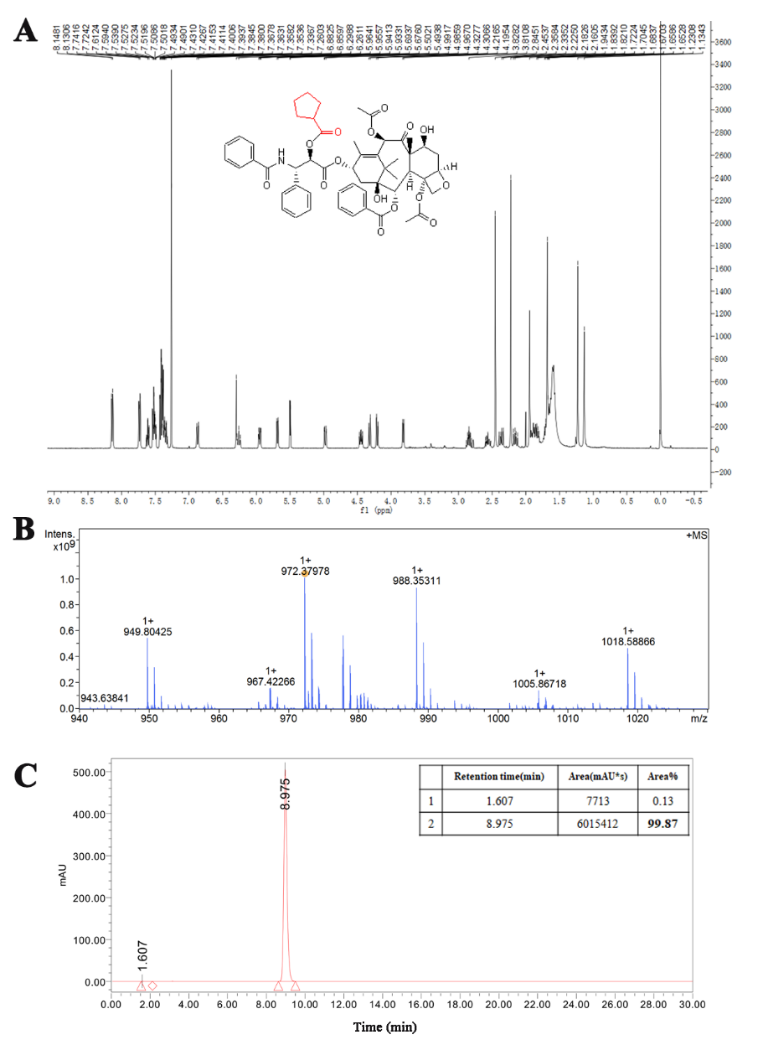
**

**Figure S4.** Characterizations of PTX-Cy-C. (A) ^1^H NMR spectrum. (B) Mass spectrum. (C) The purity.

^1^H NMR (400 MHz, Chloroform-d) δ 8.14 (d, 2H, o-2O-ArH), 7.73 (d, 2H, o-3’NH-ArH), 7.64 - 7.58 (m, 1H, p-2O-ArH), 7.55 - 7.31 (m, 10H, 3’-ArH and 2O-ArH and 3’NH-ArH), 6.87 (d, 1H, 3’-NH-), 6.30 (s, 1H, 10-H), 6.26 (t, 1H, 13-H), 5.95 (dd, 1H, 3’-H), 5.68 (d, 1H, 2-H), 5.50 (d, 1H, 2’-H), 4.98 (dd, 1H, 5-H), 4.45 (dd, 1H, 7-H), 4.32 (d, 1H, 20-HH), 4.21 (d, 1H, 20-HH), 3.82 (d, 1H, 3-H), 2.84 (m, 1H, OOCCHCH_2_), 2.61 - 2.52 (m, 1H, 6-HH), 2.47 (s, 4H, 7-OH, 4O-Ac-H), 2.37 (dd, 1H, 14-H), 2.23 (s, 3H, 10O-Ac-H), 2.18 - 2.11 (m, 1H, 14-H), 1.94 (s, 3H, 18-CH_3_), 1.92 - 1.80 (m, 4H, 6-HH, OOCCHCH_2_CH_2_CH_2_CH_2_), 1.75 - 1.63 (m, 9H, OOCCHCH_2_CH_2_CH_2_CH_2_, 1-OH, 19-CH_3_), 1.23 (s, 3H, 17-CH_3_), 1.13 (s, 3H, 16-CH_3_).

MS (ESI) m/z for C_53_H_59_NO_15_Na [M+Na]^+^: 972.37978.


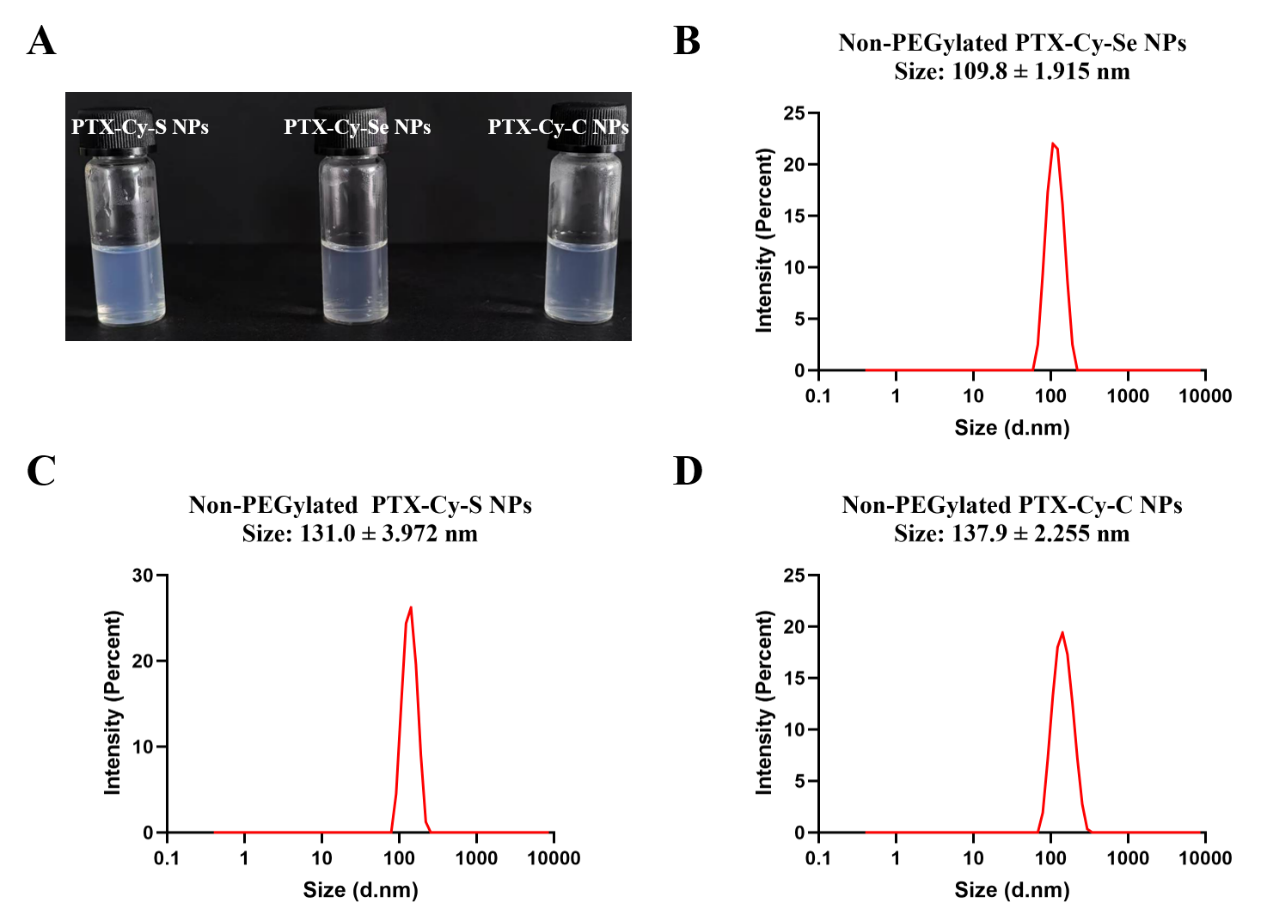


**Figure S5.** (A) The appearance of non-PEGylated prodrug nanoassemblies. (B-D) Particle size distribution of non-PEGylated PTX-Cy-Se NPs, PTX-Cy-S NPs and PTX-Cy-C NPs (0.2 mg mL^-1^).


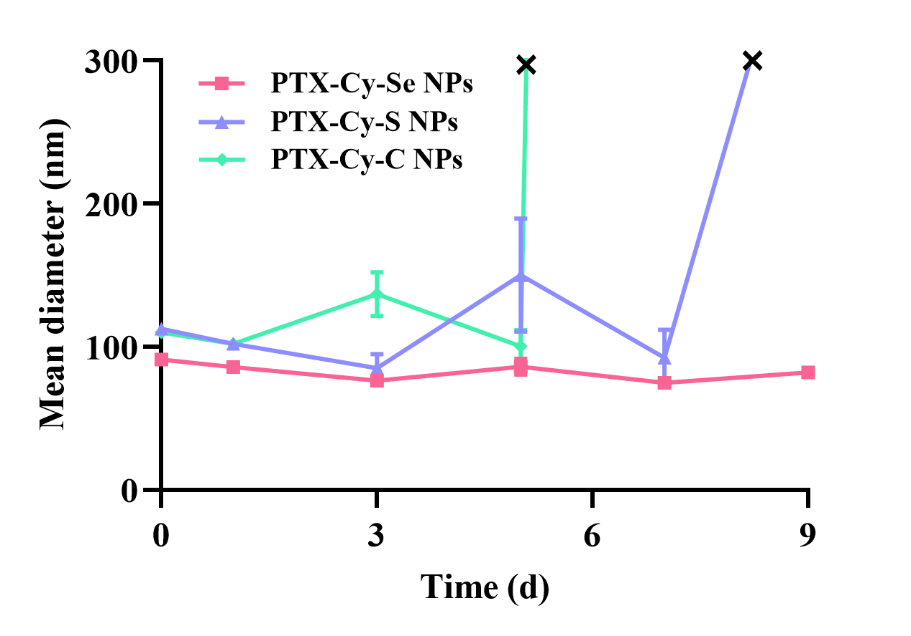


**Figure S6.** Stability of non-PEGylated prodrug nanoassemblies at room temperature for 9 days.


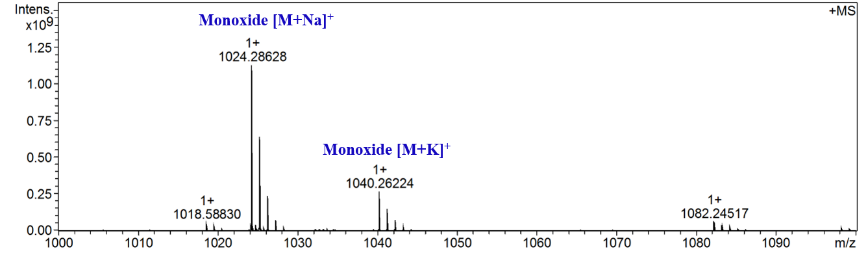


**Figure S7.** Mass spectrum of the oxidation intermediate of PTX-Cy-S NPs.

**
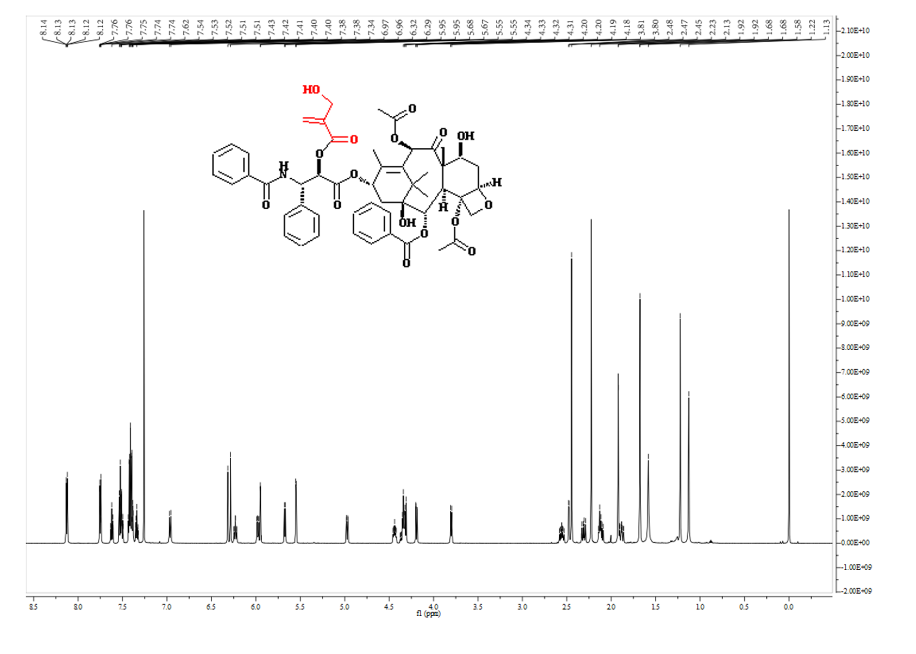
**

**Figure S8.** ^1^H NMR spectrum of the oxidation intermediate of PTX-Cy-Se NPs.


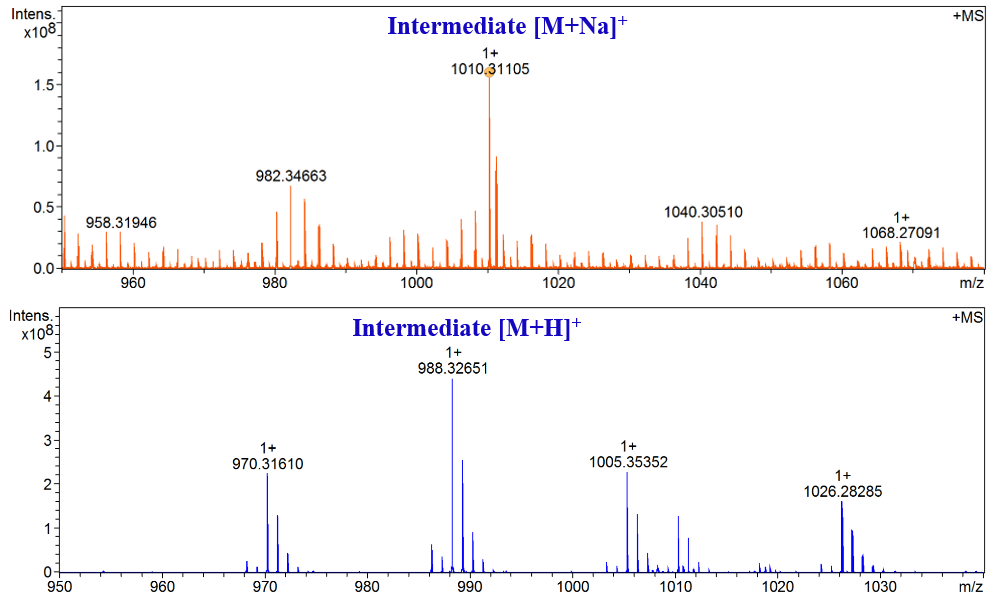


**Figure S9.** Mass spectra of the reduction intermediate of PTX-Cy-S NPs.


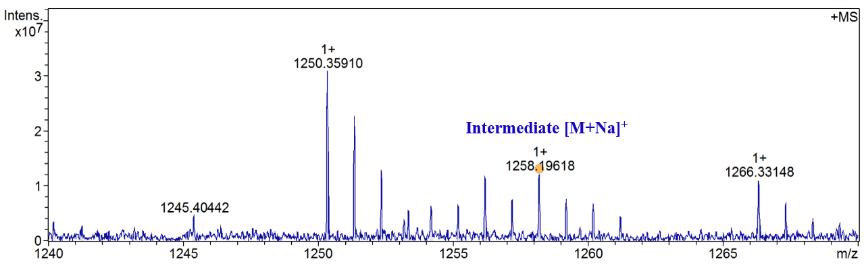


**Figure S10.** Mass spectrum of the reduction intermediate of PTX-Cy-Se NPs.


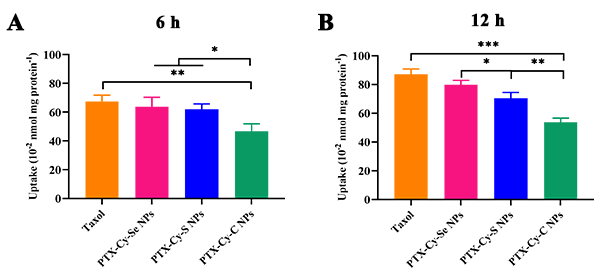


**Figure S11.** Release of PTX after uptake of prodrug nanoassemblies by A549 cells.


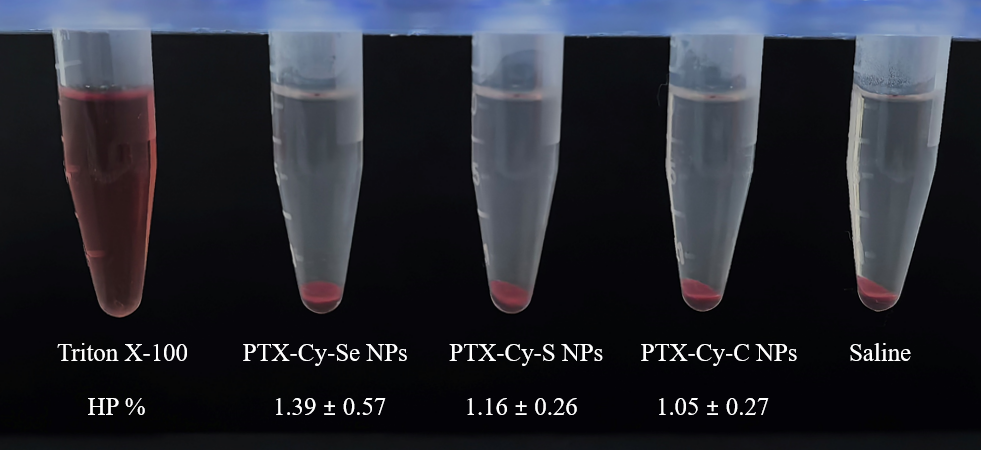


**Figure S12.** Hemolysis experiments and hemolysis percentage of prodrug nanoassemblies.

**
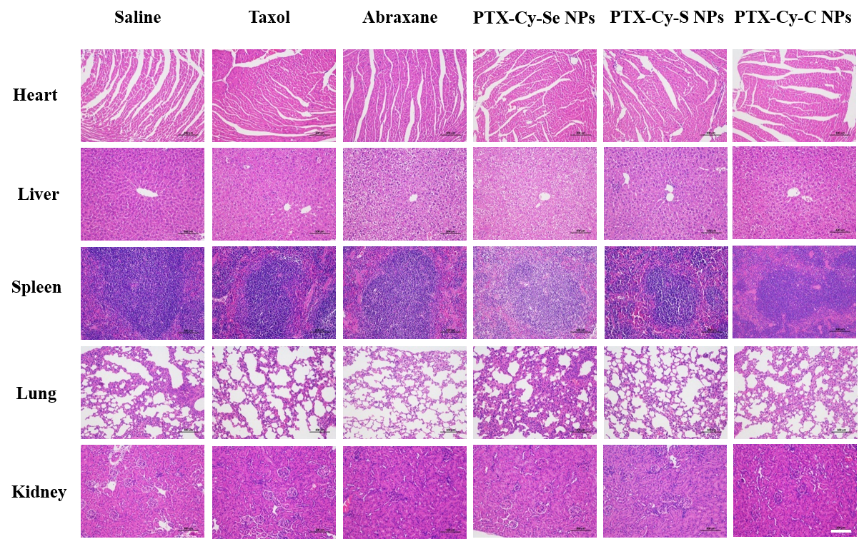
**

**Figure S13.** H&E staining of the Lewis tumor sections. Scale bar: 100 μm.

**
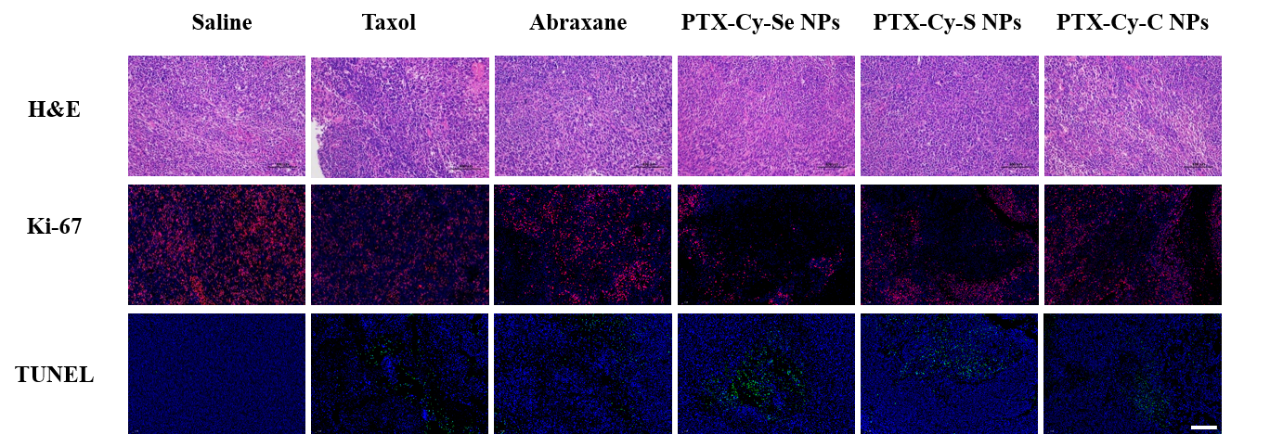
**

**Figure S14.** Tumor tissue staining of the Lewis tumor sections. Scale bar: 100 μm.

**
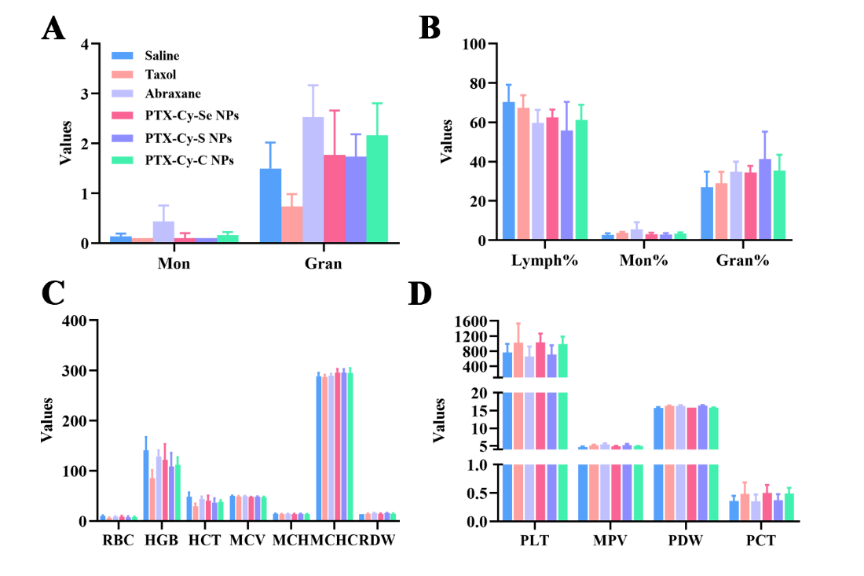
**

**Figure S15.** Hematologic analysis of Lewis tumor-bearing mice. (A) Mon: monocyte count (10^9^ L^-1^), Gran: granulocyte count (10^9^ L^-1^). (B) Lymph%: lymphocyte percentage (%), Mon%: monocyte percentage (%), Gran%: granulocyte percentage (%). (C) RBC: red blood cell count (10^12^ L^-1^), HGB: **hemoglobin (**g L^-1^)**,** HCT: **hematocrit (%),** MCV: mean red blood cell volume (fL), MCH: mean corpuscular hemoglobin (pg), MCHC: mean corpuscular hemoglobin concentration **(**g L^-1^), RDW: red cell distribution width (%). **(D) PLT: platelet count** (10^9^ L^-1^)**,** MPV: average platelet volume (fL), PDW: platelet distribution width, PCT: platelet hematocrit (%).

**
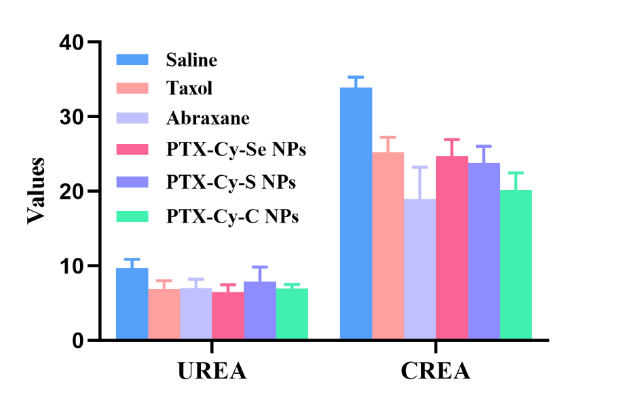
**

**Figure S16.** Hepatorenal function parameters of Lewis tumor-bearing mice. UREA: urea (mmol L^-1^), CREA: creatinine (μmol L^-1^).


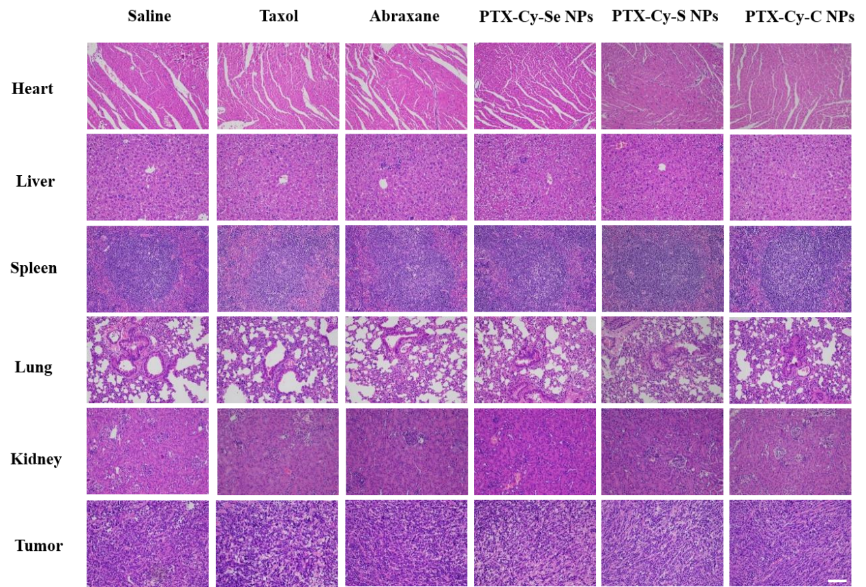


**Figure S17.** H&E staining of heterotopic 4T1 tumor sections. Scale bar: 100 μm.


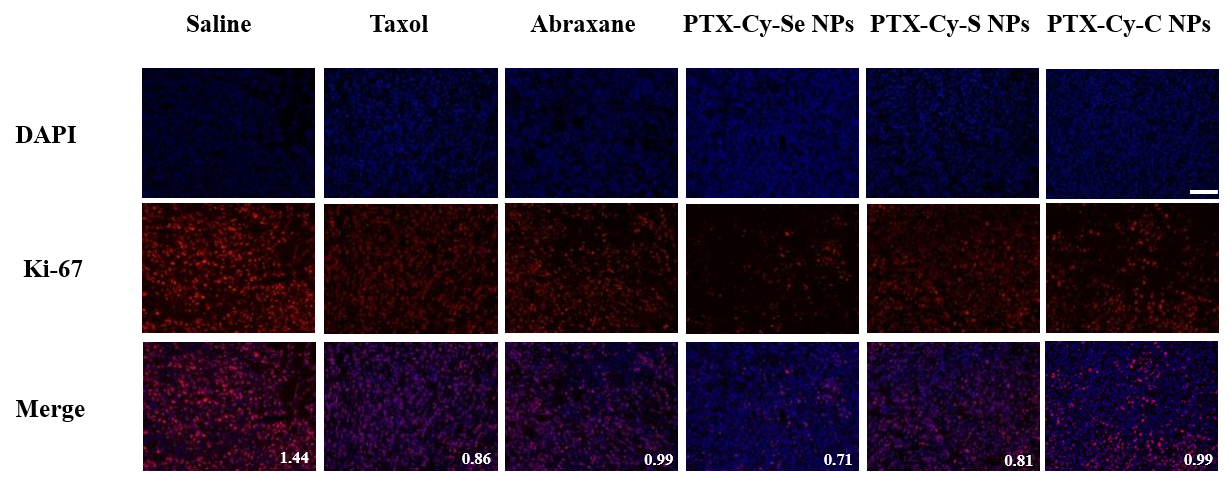


**Figure S18.** Ki-67 staining of heterotopic 4T1 tumor sections. Scale bar: 100 μm.

**
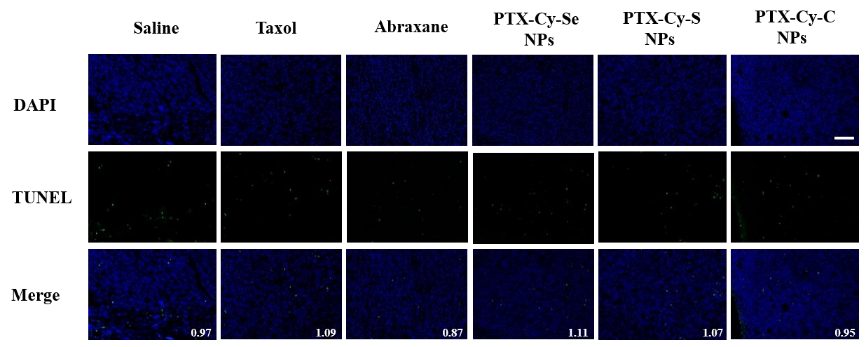
**

**Figure S19.** TUNEL staining of heterotopic 4T1 tumor sections. Scale bar: 100 μm.


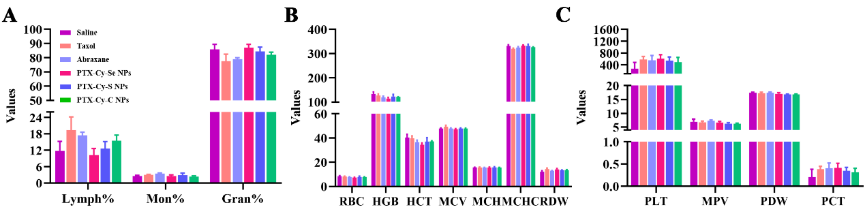


**Figure S20.** Hematologic analysis of heterotopic 4T1 tumor-bearing mice. (A) Lymph%: lymphocyte percentage (%), Mon%: monocyte percentage (%), Gran%: granulocyte percentage (%). (B) RBC: red blood cell count (10^12^ L^-1^), HGB: **hemoglobin (**g L^-1^)**,** HCT: **hematocrit (%),** MCV: mean red blood cell volume (fL), MCH: mean corpuscular hemoglobin (pg), MCHC: mean corpuscular hemoglobin concentration **(**g L^-1^), RDW: red cell distribution width (%). **(C) PLT: platelet count** (10^9^ L^-1^)**,** MPV: average platelet volume (fL), PDW: platelet distribution width, PCT: platelet hematocrit (%).


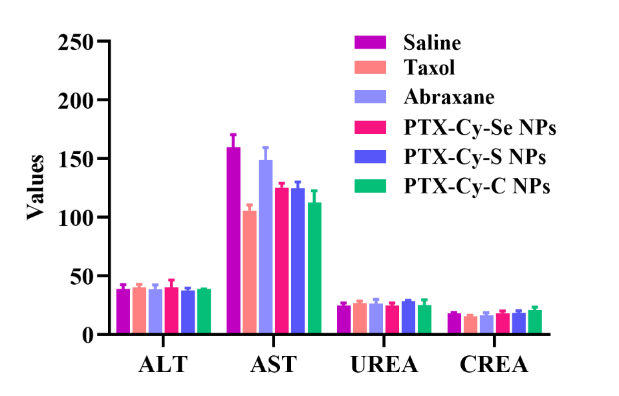


**Figure S21.** Hepatorenal function parameters of heterotopic 4T1 tumor-bearing mice. AST: aspartate aminotransferase (U L^−1^), ALT: alanine aminotransferase (U L^−1^), UREA: urea (mmol L^−1^), CREA: creatinine (μmol L^−1^).


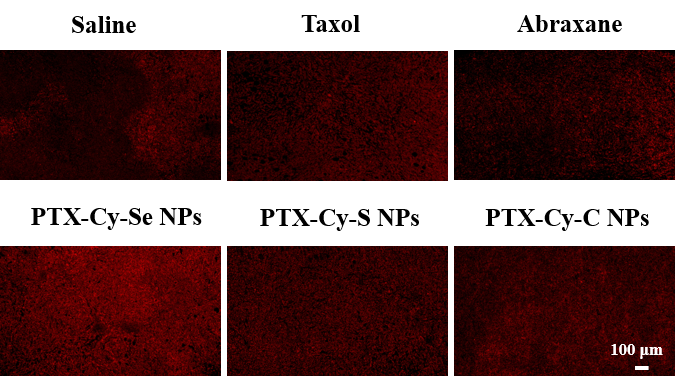


**Figure S22.** NOX4 staining of orthotopic 4T1 tumor sections. Scale bar: 100 μm.


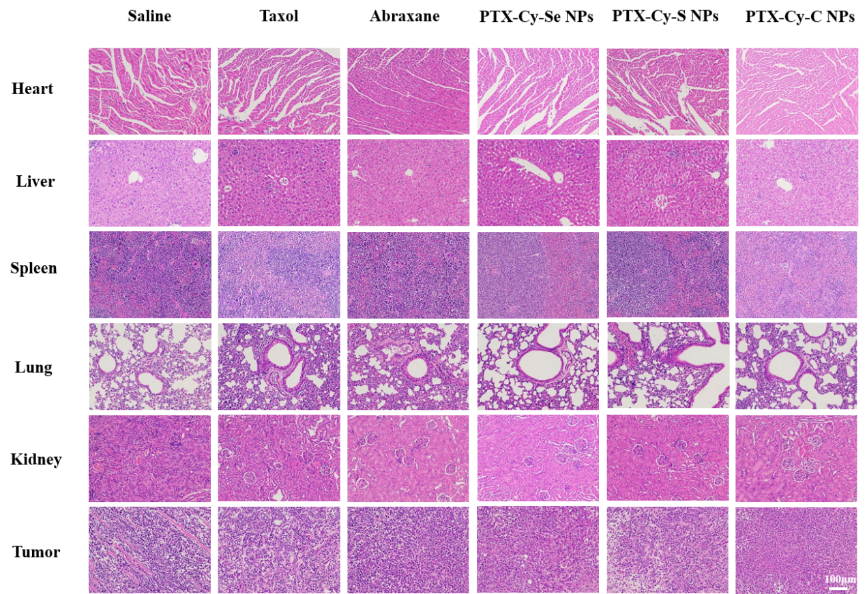


**Figure S23.** H&E staining of orthotopic 4T1 tumor sections. Scale bar: 100 μm.


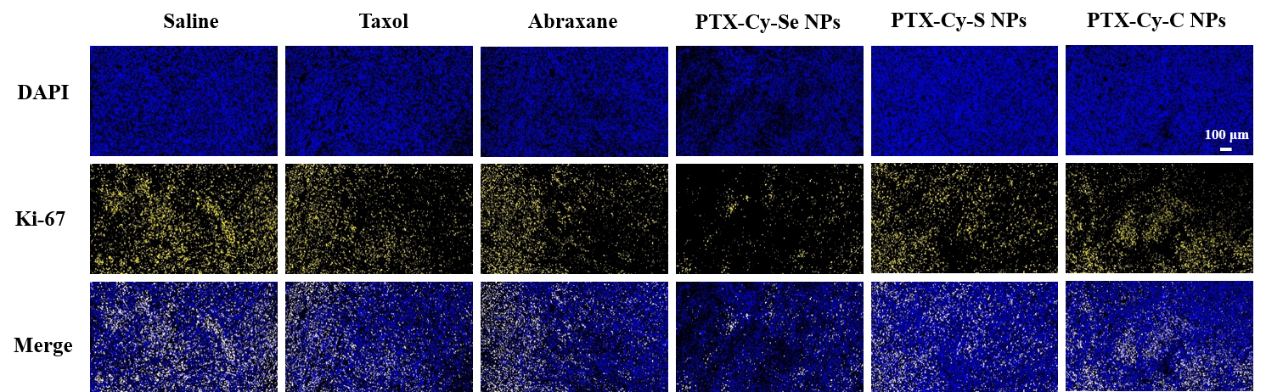


**Figure S24.** Ki-67 staining of orthotopic 4T1 tumor sections. Scale bar: 100 μm.


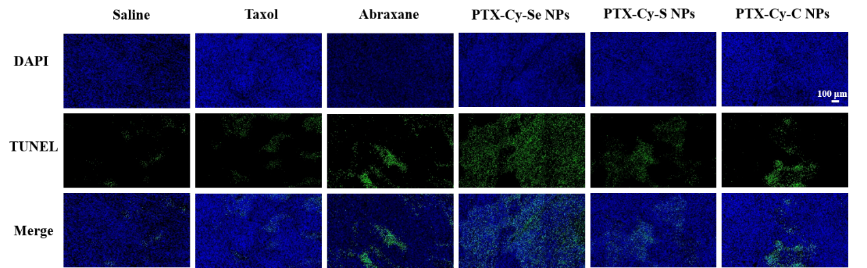


**Figure S25.** TUNEL staining of orthotopic 4T1 tumor sections. Scale bar: 100 μm.


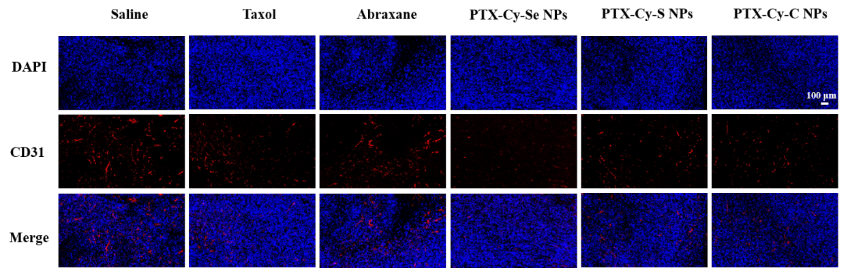


**Figure S26.** CD31 staining of orthotopic 4T1 tumor sections. Scale bar: 100 μm.


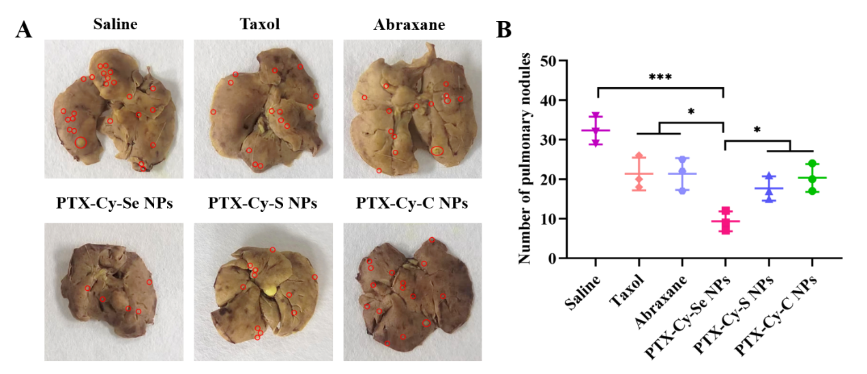


**Figure S27.** Lung metastasis in the orthotopic 4T1 tumor model.


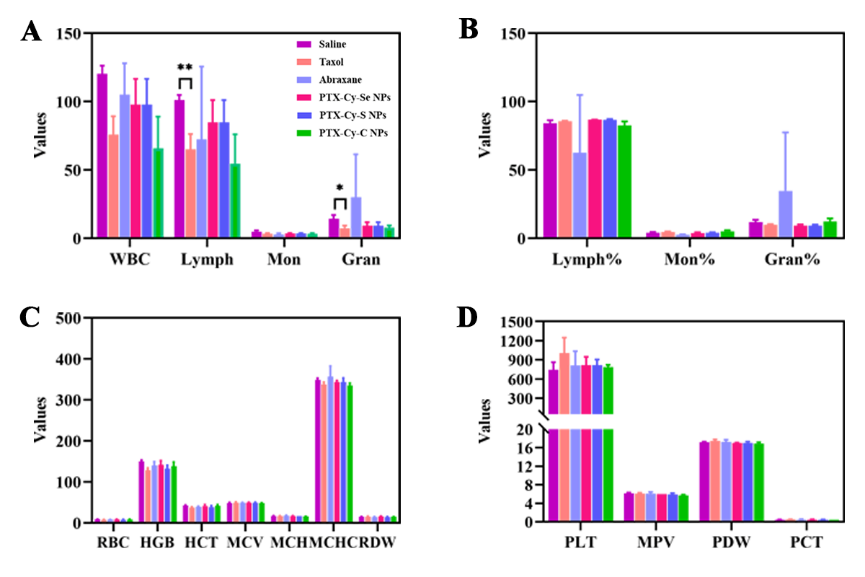


**Figure S28.** Hematologic analysis of orthotopic 4T1 tumor-bearing mice. (A) WBC: white blood cell count (10^9^ L^-1^), Lymph: lymphocyte count (10^9^ L^-1^), Mon: monocyte count (10^9^ L^-1^), Gran: granulocyte count (10^9^ L^-1^). (B) Lymph%: lymphocyte percentage (%), Mon%: monocyte percentage (%), Gran%: granulocyte percentage (%). (C) RBC: red blood cell count (10^12^ L^-1^), HGB: **hemoglobin (**g L^-1^)**,** HCT: **hematocrit (%),** MCV: mean red blood cell volume (fL), MCH: mean corpuscular hemoglobin (pg), MCHC: mean corpuscular hemoglobin concentration **(**g L^-1^), RDW: red cell distribution width (%). **(D) PLT: platelet count** (10^9^ L^-1^)**,** MPV: average platelet volume (fL), PDW: platelet distribution width, PCT: platelet hematocrit (%).


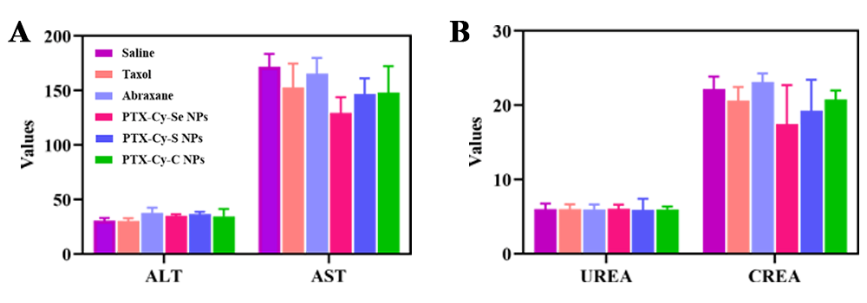


**Figure S29.** Hepatorenal function parameters of orthotopic 4T1 tumor-bearing mice. (A) AST: aspartate aminotransferase (U L^−1^), ALT: alanine aminotransferase (U L^−1^). (B) UREA: urea (mmol L^−1^), CREA: creatinine (μmol L^−1^).

**Supplementary Tables**

**Table S1.** Characterization of non-PEGylated prodrug nanoassemblies (0.2 mg mL^-1^).

| **Nanoassemblies** | **Size (nm)** | **PDI** | **Zeta potential (mV)** |
| --- | --- | --- | --- |
| **PTX-Cy-Se NPs** | 109.8 ± 1.915 | 0.086 ± 0.033 | -17.9 ± 0.93 |
| **PTX-Cy-S NPs** | 131.0 ± 3.972 | 0.118 ± 0.051 | -20.3 ± 2.22 |
| **PTX-Cy-C NPs** | 137.9 ± 2.255 | 0.147 ± 0.071 | -13.6 ± 0.98 |

**Table S2.** Characterization of prodrug nanoassemblies (1 mg mL^-1^).

| **Nanoassemblies** | **Size (nm)** | **PDI** | **Zeta potential (mV)** | **DL (w/w, %)** |
| --- | --- | --- | --- | --- |
| **PTX-Cy-Se NPs** | 119.2 ± 4.400 | 0.062 ± 0.048 | -20.0 ± 0.61 | 63.18 |
| **PTX-Cy-S NPs** | 124.5 ± 2.081 | 0.163 ± 0.050 | -21.4 ± 0.84 | 69.33 |
| **PTX-Cy-C NPs** | 130.8 ± 2.326 | 0.051 ± 0.021 | -21.0 ± 1.20 | 71.95 |

**Table S3.** IC_50_ values (nM) of Taxol and prodrug nanoassemblies.

| **Cell lines** | **Taxol** | **PTX-Cy-Se NPs** | **PTX-Cy-S NPs** | **PTX-Cy-C NPs** |
| --- | --- | --- | --- | --- |
| **4T1** | 10.02 ± 0.71 | 48.08 ± 13.76 | 86.09 ± 21.86 | 219.40 ± 42.09 |
| **A549** | 30.93 ± 9.43 | 46.08 ± 19.34 | 75.41 ± 15.39 | 173.70 ± 58.05 |
| **B16-F10** | 14.89 ± 1.19 | 21.61 ± 7.12 | 30.85 ± 9.02 | 53.12 ± 10.65 |
| **3T3** | 38.58 ± 7.92 | 427.70 ± 22.30 | 572.00 ± 43.49 | 850.40 ± 134.6 |

**Table S4.** The selectivity index (SI) of Taxol and prodrug nanoassemblies.

| **Cell lines** | **Taxol** | **PTX-Cy-Se NPs** | **PTX-Cy-S NPs** | **PTX-Cy-C NPs** |
| --- | --- | --- | --- | --- |
| **4T1** | 3.85 | 8.90 | 6.64 | 3.88 |
| **A549** | 1.25 | 9.28 | 7.59 | 4.90 |
| **B16-F10** | 2.59 | 19.79 | 18.54 | 16.00 |

**Table S5.** Pharmacokinetic parameters of DiR and DiR-labeled prodrug nanoassemblies.

| **Formulations** | **AUC_0-24 h_ (nmol h mL^-1^)** | **C_max_ (nmol mL^-1^)** |
| --- | --- | --- |
| **DiR Solution** | 1.83 ± 0.04 | 0.11 ± 0.01 |
| **PTX-Cy-Se NPs** | 20.75 ± 2.80 | 7.29 ± 0.25 |
| **PTX-Cy-S NPs** | 19.93 ± 5.56 | 5.80 ± 0.40 |
| **PTX-Cy-C NPs** | 4.21 ± 0.64 | 1.54 ± 0.05 |
